# ELF4/TRIB3/CDK6 Axis Promotes Cancer Stem Cell Activity in Endometrial Cancer

**DOI:** 10.1002/jcp.70113

**Published:** 2025-11-25

**Authors:** Chun‐Yu Chen, Yueh‐Chun Lee, Yu‐Hao Huang, Wen‐Ling Wang, Wen‐Wei Chang

**Affiliations:** ^1^ Department of Emergency Medicine Tungs' Taichung MetroHarbor Hospital Taichung Taiwan; ^2^ Post‐Baccalaureate Medicine, National Chung Hsing University Taichung Taiwan; ^3^ Department of Radiation Oncology Chung Shan Medical University Hospital Taichung Taiwan; ^4^ School of Medicine Chung Shan Medical University Taichung Taiwan; ^5^ Department of Biomedical Sciences Chung Shan Medical University Taichung Taiwan; ^6^ Department of Medical Research Chung Shan Medical University Hospital Taichung Taiwan

**Keywords:** cancer stem cells, cyclin‐dependent kinase 6, E74‐like ETS transcription factor 4, endometrial cancer, tribbles pseudokinase 3

## Abstract

Endometrial cancer (EC) is the most prevalent gynecological malignancy globally. Here, we explored the role of E74‐like ETS transcription factor 4 (ELF4) in EC progression. Using the TISIDB web tool to analyze TCGA data, we found that elevated ELF4 expression correlates with higher histological grades and reduced overall survival in EC patients. Tissue microarray analysis confirmed a grade‐dependent increase in ELF4 protein levels. Knockdown of ELF4 in EC cell lines (AN3CA, HEC‐1A) and patient‐derived EC cells suppressed proliferation, cell cycle progression, and cancer stem cell (CSC) activity. Database analysis and RNA interference identified cyclin‐dependent kinase 6 (CDK6) as a downstream target of ELF4. ELF4 silencing reduced CDK6 mRNA and protein expression, while chromatin immunoprecipitation revealed direct binding of ELF4 to the CDK6 promoter. Conversely, ELF4 overexpression upregulated CDK6. Knockdown of CDK6 or treatment with the CDK4/6 inhibitor Palbociclib diminished tumorsphere formation and expression of stemness markers (OCT4, NANOG, c‐MYC) in both conventional and patient‐derived EC cells. We previously reported that the tribbles pseudokinase 3 (TRIB3)/ELF4 complex transactivates CTNNB1 expression; here, we show that TRIB3 knockdown also downregulates CDK6 at mRNA and protein levels, suggesting cooperative regulation of CDK6 by ELF4 and TRIB3. In EC specimens, ELF4, TRIB3, and CDK6 expression positively correlated, and Kaplan‐Meier analysis indicated that high co‐expression of these genes predicted the poorest overall survival. Collectively, our findings establish the ELF4/TRIB3/CDK6 axis as a critical regulator of EC progression and CSC maintenance, highlighting its potential as a therapeutic target for EC.

## Graphical abstract

ELF4 can complex with TRIB3 to promote CDK6 expression, which is involved in EC cell proliferation and cancer stemness activities. Inhibition of CDK6 significantly represses EC‐CSC activities by downregulating c‐MYC, OCT4 and NANOG.

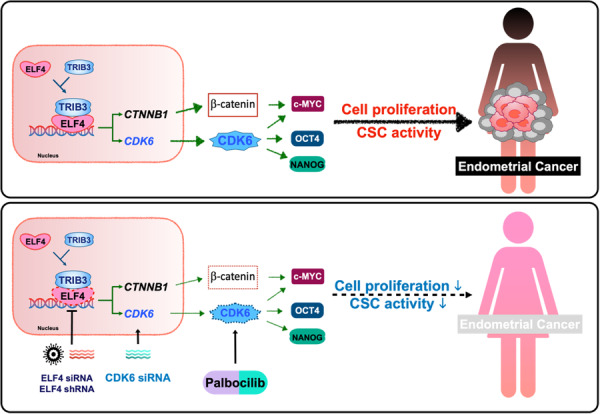

## 1 Introduction

Endometrial cancer (EC), the most common gynecologic malignancy, originates from the uterine inner epithelial lining and is marked by rising incidence and mortality, with 417,336 new cases and nearly 100,000 deaths worldwide in 2020 (Makker et al. 2021). It ranks as the sixth most frequent cancer among women, with the highest burden in North America and Western Europe (Siegel et al. 2023). EC is categorized into type I (endometrioid, estrogen‐driven) and type II (non‐endometrioid, high‐grade) histological subtypes, and four molecular subtypes per The Cancer Genome Atlas: ultramutated POLE (POLEmut), hypermutated microsatellite instability (MSI‐H/MMRd), copy‐number high (p53abn), and copy‐number low (NSMP/p53wt), which influence metastasis and recurrence (Makker et al. 2021; Njoku et al. 2022; Restaino et al. 2023; Soslow et al. 2019). Despite advances in surgery, chemotherapy, and radiation, advanced or recurrent EC cases (stages III–IV) exhibit a 5‐year survival rate of 15%–17%, driven by poor treatment response and resistance (Chambers 2022). Emerging therapies like lenvatinib plus pembrolizumab lack specificity for cancer stem cells (CSCs), a critical gap given their role in recurrence and resistance (Banz‐Jansen et al. 2022; Mitamura et al. 2019). In EC, CSCs (EC‐CSCs) fuel tumor initiation, growth, invasion, metastasis, and relapse through self‐renewal and differentiation (Fraszczak and Barczynski 2024; Giannone et al. 2019). The WNT/β‐catenin pathway, often hyperactive in EC‐CSCs, upregulates stemness markers such as β‐catenin, c‐MYC, NANOG, and OCT4, perpetuating tumor progression (Banz‐Jansen et al. 2022; Giannone et al. 2019). Thus, targeting EC‐CSCs and their associated signaling pathways offers a promising strategy to halt progression and prevent recurrence.

E74‐like ETS transcription factor 4 (ELF4), a member of the ETS family, regulates diverse physiological processes, including tumorigenesis and stem cell quiescence (Suico et al. 2017). As a transcriptional activator, ELF4 interacts with partner proteins to modulate gene expression (Suico et al. 2017). For example, it binds the oncofusion protein AML1/ETO in acute myeloid leukemia to regulate hematopoietic stem cell quiescence (Lacorazza et al. 2006) and cooperates with RUNX2 to drive osteoblast differentiation (Lee et al. 2010). Our prior work demonstrated that tribbles pseudokinase 3 (TRIB3) partners with ELF4 to upregulate β‐catenin expression, promoting EC development (Wang et al. 2020). Aberrant ELF4 expression is implicated in cancer cell proliferation, stemness, and therapy resistance across multiple malignancies, including leukemia (Sashida et al. 2009), ovarian cancer (Yao et al. 2007), glioma (Bazzoli et al. 2012), cervical cancer (Guo et al. 2019), hepatocellular carcinoma (Sze et al. 2021), and colorectal cancer (Chen et al. 2023). Despite these insights, ELF4's specific contributions to EC progression and its underlying mechanisms remain poorly defined, warranting further investigation.

Cyclin‐dependent kinase 6 (CDK6) is a critical regulator of cell cycle progression, promoting proliferation by phosphorylating retinoblastoma protein in complex with cyclins (Fassl et al. 2022). Elevated CDK6 expression has emerged as a prognostic biomarker and predictor of chemosensitivity in various cancers, including EC (Ikeda et al. 2015). Beyond its kinase activity, CDK6 exerts kinase‐independent effects, enhancing angiogenesis (Kollmann et al. 2019), stem cell activation (Li et al. 2019; Uras et al. 2019), and epithelial‐mesenchymal transition (EMT)(J. Li et al. 2019). These multifaceted roles position CDK6 as a promising target for antitumor therapy. CDK4/6 inhibitors—such as Palbociclib, Abemaciclib, and Ribociclib—have been approved by the US Food and Drug Administration (FDA) for treating metastatic HR + /HER2‐ breast cancer (Cardoso et al. 2018; Fassl et al. 2022), demonstrating efficacy in inhibiting tumor cell proliferation, migration, and invasion (Goel et al. 2017; Hu et al. 2020). Despite CDK6's established oncogenic potential and therapeutic relevance across cancer types, its regulatory mechanisms and specific contributions to EC progression remain poorly understood, necessitating further investigation.

This study investigates the clinical significance and mechanisms of ELF4 in EC, focusing on its interplay with CDK6 and TRIB3 in EC‐CSCs. We demonstrate that ELF4 directly upregulates CDK6 expression by binding its promoter, with elevated CDK6 levels linked to clinicopathological features of EC. Downregulation of CDK6 inhibits CSC activity, highlighting the ELF4‐CDK6‐TRIB3 axis as a potential therapeutic target to curb EC progression and recurrence.

## 2 Materials and Methods

### 2.1 Cell Culture

Human endometrial cancer (EC) cell lines AN3CA and HEC‐1A were obtained from the American Type Culture Collection (Manassas, VA, USA). EMC4, EMC5, and EMC6 EC cell lines were established from Taiwanese patient specimens from our previous study (Wang et al. 2020). AN3CA, EMC5, and EMC6 cells were cultured in DMEM (Gibco, Grand Island, NY, USA), while HEC‐1A cells were grown in McCoy's 5A medium (Gibco), both supplemented with 10% FBS (Hyclone, South Logan, UT, USA) and 1% penicillin–streptomycin–amphotericin B (Gibco) at 37°C with 5% CO₂ in a humidified incubator. Cell lines were authenticated by STR profiling and confirmed mycoplasma‐free.

### 2.2 Database Analysis

Gene expression levels of ELF4 and CDK6 were validated using Gene Expression Profiling Interactive Analysis 2 (GEPIA2) (Tang et al. 2019) and Tumor and Immune System Interaction Database (TISIDB) (Ru et al. 2019). Transcriptomic profiles of EC were retrieved from GEO (GSE17025, GDS4589; *n* = 91 EC samples). The CDK6 promoter sequence was obtained from the Eukaryotic Promoter Database (EPD; https://epd.expasy.org/epd/). Expression and multi‐gene correlation analyses were performed in GEPIA2 using TPM (Transcripts Per Million) values from TCGA data. Kaplan‐Meier survival analysis was conducted with KM plotter (http://kmplot.com/analysis/) using the TCGA‐UCEC dataset (*n* = 548). Overall survival (OS) and recurrence‐free survival (RFS) were assessed for ELF4 and CDK6, with high‐ and low‐expression groups defined by the optimal cut‐off (lowest FDR). Log‐rank *p* < 0.05 was considered significant, with hazard ratios (HR) and 95% confidence intervals reported.

### 2.3 Cell Viability Assay

Cell viability was assessed using the MTT assay. AN3CA, HEC‐1A, and EMC6 cells were seeded at 3 × 10³ cells/well in 96‐well plates (Greiner Bio‐One, Kremsmünster, Austria) and treated with Palbociclib (MedChemExpress, Monmouth Junction, NJ, USA) for 72 h at 37°C with 5% CO₂. MTT solution (10 μL, 5 mg/mL; Thermo Fisher Scientific, Waltham, MA) was added, and cells were incubated for 2 h at 37°C. Formazan crystals were dissolved in 100 μL DMSO (Sigma‐Aldrich, St. Louis, MO, USA). Absorbance was measured at 570 nm using a BioTek Synergy H1 microplate reader (BioTek, Winooski, VT, USA).

### 2.4 Colony Formation Assay

Cells were seeded into uncoated 12‐well plates (Greiner Bio‐One) at a density of 300 cells/well in 1 mL of complete medium. AN3CA and EMC6 cells were incubated for 10 days, and HEC‐1A cells for 7 days, at 37°C in a humidified atmosphere with 5% CO₂. Colonies were fixed with 2% formaldehyde (Sigma‐Aldrich) for 20 min at room temperature, stained with 0.1% crystal violet (Sigma‐Aldrich) in 20% methanol for 1 h, and washed with distilled water to remove excess dye. Colonies (> 50 cells) were counted manually under a stereomicroscope (Leica MZ6, 10× magnification).

### 2.5 Quantitative Reverse Transcription Polymerase Chain Reaction (qRT‐PCR) Analysis

Total RNA was extracted using Trizol reagent (Thermo Fisher Scientific). Complementary DNA (cDNA) was synthesized from 1 μg RNA using the RevertAid First Strand cDNA Synthesis Kit (Thermo Fisher Scientific). Real‐time qPCR was performed using SYBR Green Master Mix (Bio‐Rad Laboratories Inc., Hercules, CA, USA) on a PCRmax Eco48 system (PCRmax, Staffordshire, UK). Cycling conditions were 95°C for 10 s and 60°C for 1 min for 40 cycles. Data were analyzed with Eco 48 software, and Ct values were determined during exponential amplification. Target gene expression was normalized to GAPDH using the 2^−ΔΔCt^ method. Primer sequences were listed in Table S1.

### 2.6 Western Blot Analysis

Cells were lysed in NETN buffer (150 mM NaCl, 20 mM Tris‐HCl pH 8.0, 1 mM EDTA, 0.5% NP‐40) with protease (MedChemExpress) and phosphatase inhibitor cocktails (MedChemExpress). Lysates (20 μg) were resolved on 10% SDS‐PAGE gels and transferred to PVDF membranes (Pall Corporation, Washington, NY, USA). Membranes were blocked with 5% skimmed milk in TBS‐T for 1 h at room temperature, then incubated with primary antibodies (1:1000 dilution) at 4°C overnight. Membranes were then incubated with HRP‐conjugated secondary antibodies (Thermo Scientific, 1:10,000) for 1 h at room temperature. Bands were visualized using a chemiluminescence substrate (Advansta Inc. San Jose, CA, USA) on an Amersham Imager 600 (GE Healthcare Life Sciences, Marlborough, MA, USA) and quantified with ImageJ. The detailed information on antibodies was listed in Table S2.

### 2.7 Small Interfering RNA (siRNA) Transfection

siRNAs targeting ELF4 (siELF4) and CDK6 (siCDK6), each consisting of a pool of three target‐specific sequences, were purchased from Santa Cruz Biotechnology (Dallas, TX, USA). A nontargeting negative control siRNA was purchased from Sigma‐Aldrich. Cells were transfected with siRNAs at final concentrations of 25–100 nM using the TransIT‐X2 Dynamic Delivery System (Mirus Bio LLC, Madison, WI, USA). After 48 h posttransfection, cells were harvested for downstream analysis, including qRT‐PCR and colony formation assays, as described in respective sections.

### 2.8 Knockdown of ELF4 and TRIB3 by Lentiviral Transduction of shRNA

Lentiviral plasmids pCMVΔ8.91 (packaging), pMD.G (envelope), and gene‐specific short hairpin RNAs (shRNAs) targeting ELF4 (shELF4#1: TRCN0000013872; shELF4#2: TRCN0000013868), TRIB3 (shTRIB3#1: TRCN0000307989; shTRIB3#2: TRCN000295920) or LacZ (shLacZ: TRCN0000231722, negative control) were obtained from the National RNAi Core Facility (Academia Sinica, Taipei, Taiwan). Lentiviruses were produced in HEK‐293T cells (ATCC, Manassas, VA, USA) by co‐transfecting with a plasmid mixture containing 2.5 μg shRNA vector, 2.25 μg pCMVΔ8.91, and 0.25 μg pMD.G using 10 μL NTRII DNA transfection reagent (T‐Pro Biotechnology, New Taipei City, Taiwan) for 48 h followed by collecting lentiviral supernatants and filtering through a 0.45 μm Syringe filter (Pall Corporation). For transduction, cells were seeded in 6‐well plates and infected at 30% confluence with lentiviruses (multiplicity of infection [MOI] of 1) in the presence of 8 μg/mL polybrene (Sigma‐Aldrich). After 18 h of transduction, the medium was replaced with fresh complete medium containing 2 μg/mL puromycin (TOKU‐E, Bellingham, WA, USA) for selection of transduced cells for 72 h, and knockdown efficiency was confirmed by qRT‐PCR or western blot.

### 2.9 Cell Cycle Analysis

Cells were trypsinized, washed twice with PBS, and fixed in 70% ethanol at −20°C for a period of 24 h. After fixation, the cells were washed once with PBS and resuspended in 300 μl of cell cycle reagent mix (20 μg/ml propidium iodide (Sigma‐Aldrich), 0.2 mg/ml RNase A (Thermo Scientific) and 0.1% Triton X‐100 (Sigma‐Aldrich) and incubated for 30 min in the dark at room temperature. The cell cycle distribution was then analyzed using a flow cytometer (FACSCanto II, BD Biosciences). The resulting data were processed and measured using FlowJo software (BD Biosciences).

### 2.10 Tumorsphere Cultivation

Single EC cells were seeded at 1 × 10^4^ cells/well in ultra‐low attachment 6‐well plates (Greiner Bio‐One) with 2 mL of tumorsphere medium: DMEM/F12 supplemented with 1% methylcellulose (Sigma‐Aldrich), 1×B‐27 Supplement (Gibco), 10 ng/mL EGF (PeproTech, Rocky Hill, NJ, USA), 10 ng/mL bFGF (PeproTech), 5 ng/mL insulin (Sigma‐Aldrich), 4 μg/mL heparin (Sigma‐Aldrich), 1 μM hydrocortisone (Sigma‐Aldrich), and 0.4% BSA (Hyclone, Logan, UT, USA) and incubated at 37°C with 5% CO₂ for 7–10 days. Tumorspheres (> 50 μm diameter) were quantified using an inverted microscope (ZEISS Vert A1, Baden‐Wurttemberg, Germany).

### 2.11 Construction of TRIB3 Fragments and Co‐Immunoprecipitation (Co‐IP)

TRIB3 full‐length (FL; amino acids 1–358) and truncated fragments (amino acids 1–180, 181–358, and 72–315) were synthesized by GENEWIZ (Azenta Life Sciences, Suzhou, China) and subcloned into the pcDNA3.0‐HA vector (Invitrogen, Carlsbad, CA, USA). The human ELF4 cDNA (RefSeq NM_001421) tagged with Myc‐DDK was obtained from OriGene Technologies (Cat. No. RC200479, Rockville, MD, USA). For cotransfection, 293 T cells were seeded at a density of 5 × 10^5^ cells per well in 6‐well plates and cultured in DMEM supplemented with 10% FBS and 1% penicillin‐streptomycin at 37°C with 5% CO_2_. After 24 h, cells were co‐transfected with 1 μg of each TRIB3‐HA construct and 1 μg of ELF4‐Myc‐DKK using the TransIT‐X2 Dynamic Delivery System (Mirus Bio) according to the manufacturer's instructions. Cells were harvested 48 h post‐transfection. For Co‐IP, cells were lysed in 200 μL RIPA buffer (50 mM Tris‐HCl pH 7.4, 150 mM NaCl, 1% NP‐40, 0.5% sodium deoxycholate, 0.1% SDS) supplemented with protease and phosphatase inhibitors (both from MedChemExpress, Monmouth Junction, NJ, USA). Lysates were clarified by centrifugation at 12,000 × *g* for 10 min at 4°C, and protein concentration was determined using the BCA assay (Pierce, Thermo Fisher Scientific, Waltham, MA, USA). Equal amounts of protein (500 μg) were incubated with 2 μg anti‐HA antibody or isotype‐matched IgG overnight at 4°C with gentle rotation. Immune complexes were precipitated with 40 μL Protein G Mag Sepharose beads (Cytiva, Marlborough, MA, USA) for 2 h at 4°C. Beads were washed four times with 1 mL IP buffer (20 mM HEPES pH7.9, 2 mM MgCl2, 0.2 mM EDTA, 0.1 mM KCl, 1 mM dithiothreitol, 10% glycerol and 0.1% NP‐40) and bound proteins were eluted by boiling in 2× sample buffer for 10 min. Samples were performed western blot analysis by using anti‐Flag or anti‐HA primary antibodies. The antibody information can be found in Table S2.

### 2.12 Immunohistochemistry (IHC) Staining

A human endometrial cancer tissue microarray (TMA; Cat. No. EM1021c) was obtained from TissueArray.Com LLC (Derwood, MD, USA), containing 97 endometrioid adenocarcinoma cases and 5 normal uterus tissues, with available data of pathology grade, TNM, and clinical stage (AJCC 8.0). IHC staining was performed as described (Huang et al. 2024). Slides were incubated overnight at 4°C with primary antibodies: anti‐ELF4 (1:100) and anti‐CDK6 (1:200). Signal was developed using an avidin‐biotin‐peroxidase method with DAB substrate (DAKO, Carpinteria, CA, USA), followed by hematoxylin counterstaining (Sigma‐Aldrich) and mounting with Permount (Merck, Darmstadt, Germany). Images were captured and analyzed using a TissueFAXS Plus system (TissueGnostics GmbH, Vienna, Austria).

### 2.13 Luciferase Reporter Assay

The CDK6 promoter (RefSeq NM_001259; −1524/+180, 1.7 kb) was synthesized and cloned into pGL3‐basic (Promega Corporation, Madison, WI, USA) by GENEWIZ (Azenta Life Sciences, Suzhou, China). Deletion constructs (−524/+180 and +8/+180) were generated by PCR and subcloned into pGL3‐basic. For performing reporter assay, EC cells were co‐transfected with 0.2 μg of CDK6 reporter constructs and 0.5 ng of pRL‐TK (Promega) using 1 μL TransIT‐X2. After 48 h, luciferase activity was measured with the Dual‐Glo system (Promega) on a GloMax 20/20 Luminometer (Promega). Relative luciferase activity (firefly/Renilla ratio) was normalized to pGL3‐basic.

### 2.14 Chromatin‐Immunoprecipitation (Ch‐IP) Assay

ChIP assays were performed as described (Wang et al. 2020) with anti‐ELF4 antibody or isotype‐matched IgG, followed by precipitation with Protein G Mag Sepharose beads (Cytiva, Marlborough, MA, USA for 2 h at 4°C. After cross‐linking and protein digestion, immunoprecipitated DNA was purified using a QIAquick PCR Purification Kit (QIAGEN, Hilden, Germany) followed by qPCR analysis of specific DNA fragments using primers listed in Table S1.

### 2.15 Statistical Analysis

Statistical analyses were conducted using GraphPad Prism 7 (GraphPad Software, San Diego, CA, USA). Differences between two groups were assessed by Student's *t* test, while multiple group comparisons used one‐way ANOVA with Tukey's post hoc test. Overall survival (OS) and recurrence‐free survival (RFS) were analyzed by the Kaplan‐Meier method with log‐rank tests in GraphPad Prism. Statistical significance was defined as *p* < 0.05. Unless stated otherwise, data represent at least two independent experiments with three technical replicates.

## 3 Results

### 3.1 Elevated ELF4 Expression Correlates With Poor Prognosis in EC

**Figure 1:**
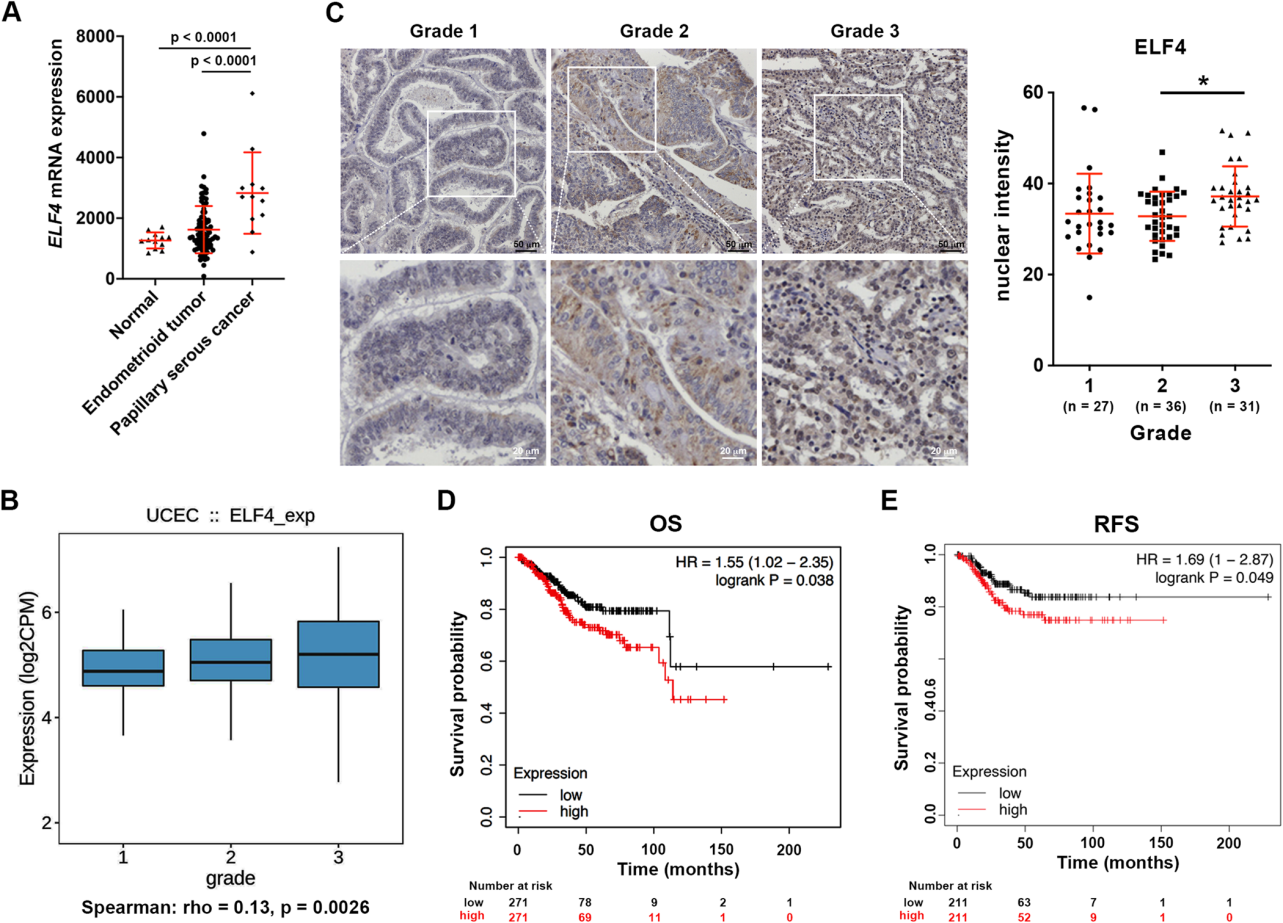
ELF4 is highly expressed in EC and is associated with poor prognosis. (A) mRNA expression of ELF4 in endometrioid tumors, papillary serous cancers, and normal endometrial tissues from GEO dataset GSE17025. Data presented as mean ± SD. The *p* values were adjusted by Tukey's method between each group. (B) Spearman correlation analysis of ELF4 expression and histological grade in TCGA Uterine Corpus Endometrial Carcinoma (UCEC) database, analyzed using TISIDB. (C) IHC analysis of ELF4 protein expression across histological grades in slides of an EC tissue microarray. Left panel: Representative images of ELF4 staining intensity in EC specimens, with insets showing magnified view of indicated area. Right panel: Quantification of ELF4 expression in human EC specimens using HistoQuest software, presented as mean ± SD; *p < 0.05 by Tukey's multiple comparisons test. (D, E) Kaplan‐Meier curves of UCEC patients from TCGA database, stratified by median ELF4 mRNA expression levels: (D) Overall survival (OS) and (E) Recurrence‐free survival (RFS). Significance determined by log‐rank test for both.

In the GEO dataset (GSE17025, GDS4589) of 103 stage I EC samples, ELF4 mRNA levels were significantly higher in endometrioid, and papillary serous tumors compared to nontumor tissues (Figure 1A). TISIDB analysis showed a positive correlation between ELF4 expression and tumor grade (Figure 1B). IHC of an EC tissue microarray revealed elevated ELF4 protein in grade 3 tumors, with representative images across grades shown in Figure 1C. Kaplan‐Meier analysis of the UCEC dataset from TCGA, using median ELF4 mRNA expression as the cutoff, indicated that high ELF4 levels were associated with shorter OS (Figure 1D) and RFS (Figure 1E) compared to low levels. These data link elevated ELF4 expression to EC progression and poor prognosis.

### 3.2 ELF4 Downregulation Suppresses Proliferation and Induces Cell Cycle Arrest in EC Cells

**Figure 2:**
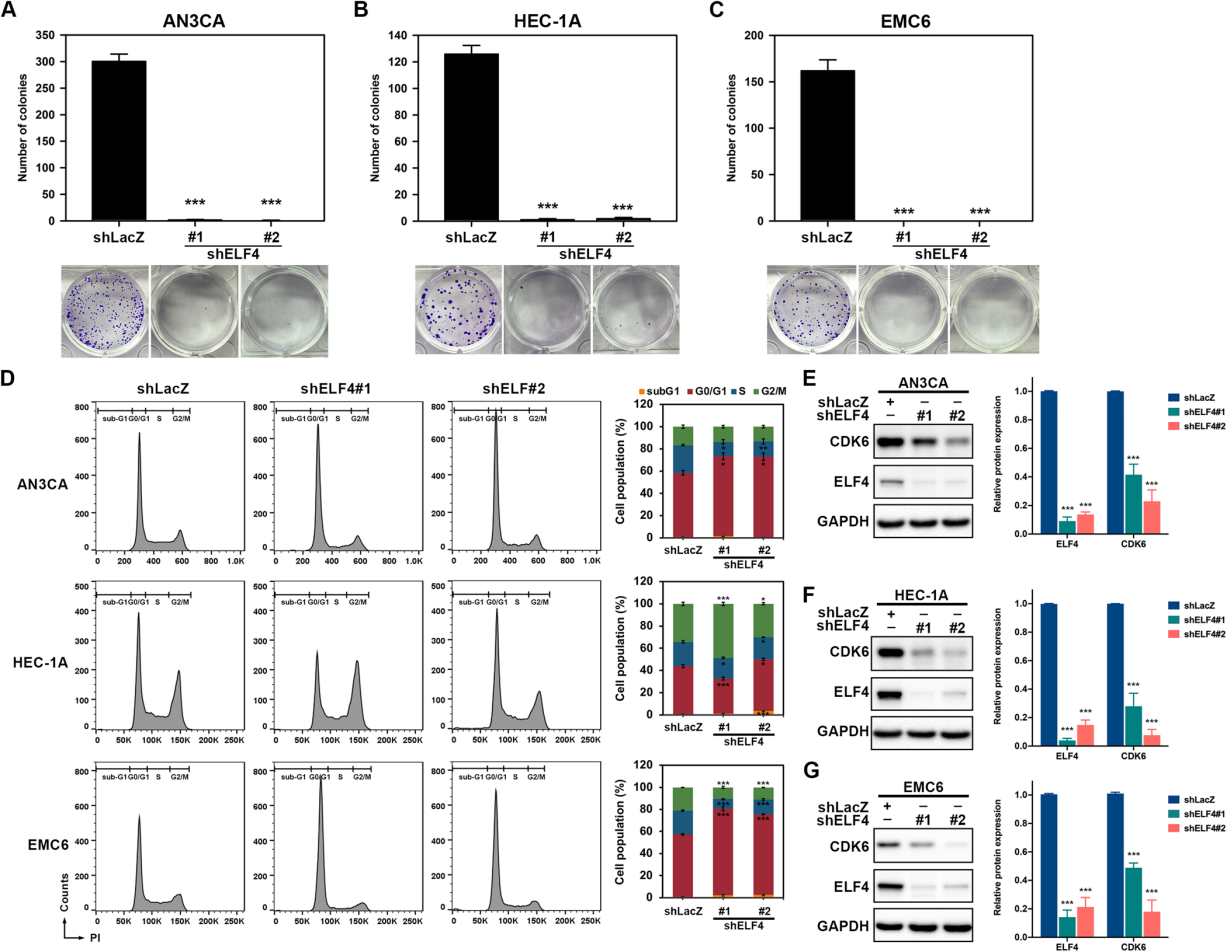
Knockdown of ELF4 suppresses cell proliferation and reduces CDK6 protein levels in EC cells. (A–C) Colony formation assay in (A) AN3CA, (B) HEC‐1A, and (C) EMC6 cells transduced with shLacZ or ELF4‐specific short hairpin RNAs (shRNAs), followed by crystal violet staining and colony counting. (D) Cell cycle analysis by propidium iodide (PI) staining and flow cytometry in cells transduced with shLacZ or ELF4‐specific shRNAs. Data are presented as mean ± SD; *p < 0.05; **p < 0.01; ***p < 0.001 versus shLacZ‐transduced cells, unpaired Student's *t* test. (E–G) Western blot analysis of ELF4 and CDK6 protein levels in (E) AN3CA, (F) HEC‐1A, and (G) EMC6 cells transduced with shLacZ or ELF4‐specific shRNAs, using GAPDH as loading control. Band intensities were quantified and expressed as mean ± SEM; ***p < 0.001 versus shLacZ, unpaired Student's *t* test.

The expression of ELF4 was silenced by using shRNAs in EC cell lines of AN3CA and HEC‐1A, and in patient‐derived EMC6 cells and confirmed by qRT‐PCR (Figure S1). Colony formation assays showed that ELF4 silencing markedly decreased colony numbers in all three cell lines (Figure 2A–C), indicating suppressed proliferation. Cell cycle analysis revealed that ELF4 knockdown increased the G0/G1 phase population while reducing S and G2/M phase fractions in AN3CA and EMC6 cells (Figure 2D, upper and lower panels). In HEC‐1A cells, shRNA#1 elevated G2/M phase cells and decreased G0/G1 and S phase proportions (Figure 2D, middle panel). As CDKs regulate cell cycle progression (Nebenfuehr et al. 2020), we examined the effect of ELF4 on CDK expression. Knockdown of ELF4 significantly reduced CDK6 protein levels in AN3CA (Figure 2E), HEC‐1A (Figure 2F), and EMC6 (Figure 2G) cells. These results suggest that ELF4 knockdown inhibits EC cell proliferation by inducing cell cycle arrest and reducing CDK6 expression.

### 3.3 ELF4 Enhances CDK6 Expression as a Key Downstream Effector in EC

**Figure 3:**
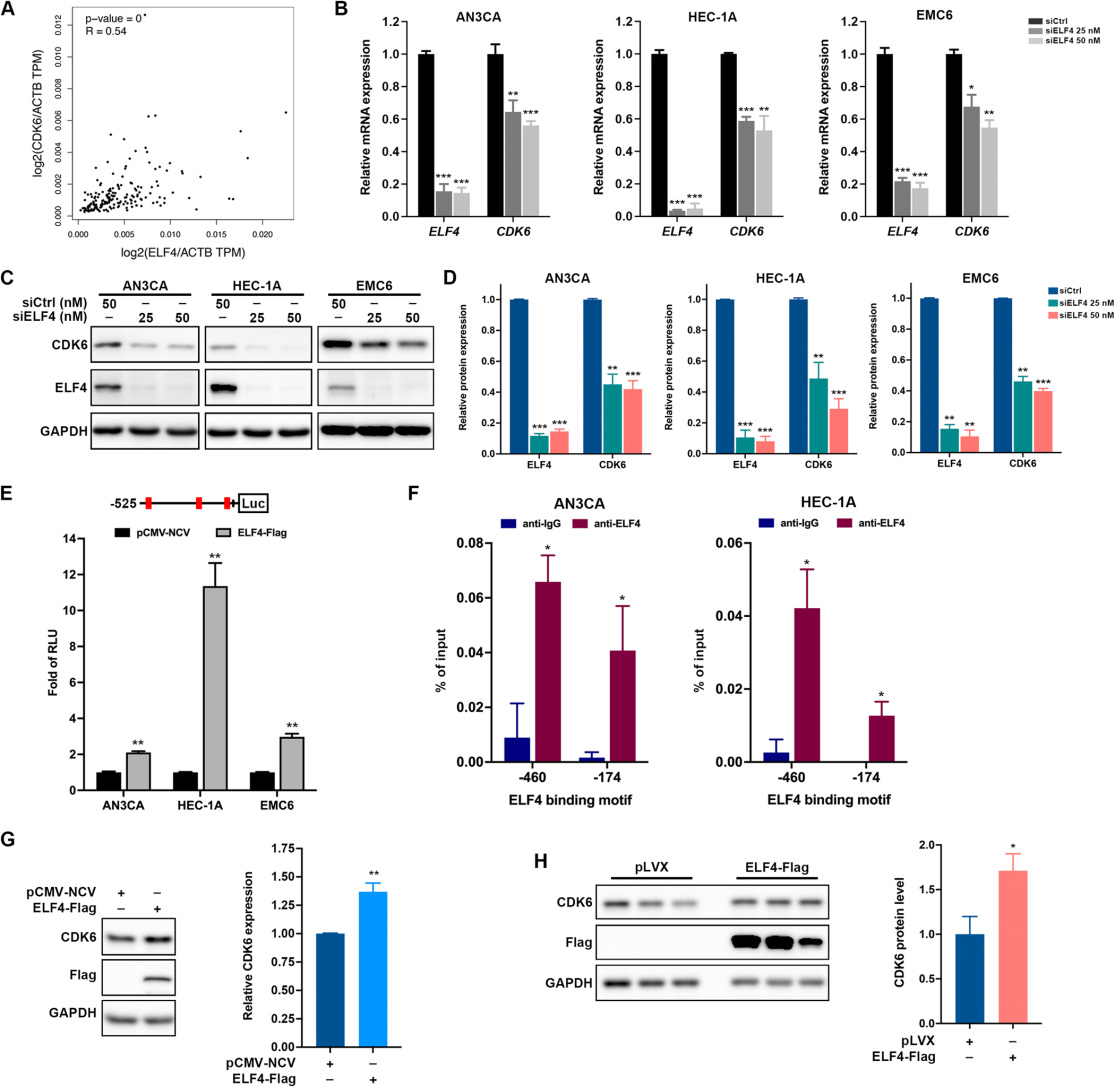
ELF4 transcriptionally regulates CDK6 expression in EC cells. (A) Spearman's correlation analysis of ELF4 and CDK6 expression in UCEC patients, performed using GEPIA2. (B) Relative mRNA levels of ELF4 and CDK6 in AN3CA, HEC‐1A, and EMC6 cells transfected with control (siCtrl) or ELF4‐specific small interfering RNAs (siELF4), measured by SYBR‐Green‐based quantitative reverse transcription PCR (qRT‐PCR). Data are fold changes (mean ± SD) versus siCtrl; *p < 0.05; **p < 0.01; ***p < 0.001, unpaired Student's *t*‐test. (C, D) Western blot analysis of CDK6, ELF4, and GAPDH expression in AN3CA, HEC‐1A, and EMC6 cells transfected with siCtrl or siELF4, using GAPDH as loading control. Band intensities quantified by ImageJ were presented as mean ± SEM (D); **p < 0.01; ***p < 0.001 versus siCtrl, Student's *t* test. (E) Upper panel: CDK6 promoter with putative ELF4 binding motifs (red bars). Lower panel: Luciferase reporter assay in AN3CA, HEC‐1A, and EMC6 cells co‐transfected with pGL3‐CDK6 promoter constructs and pCMV‐NCV or ELF4‐Flag plasmids. Data were presented as fold changes (mean ± SD) versus pCMV‐NCV; **p < 0.01, unpaired Student's *t*‐test. (F) Chromatin immunoprecipitation (ChIP) assay in AN3CA and HEC‐1A cells using anti‐ELF4 antibody or IgG control, followed by qPCR detection of the ELF4 binding fragments within CDK6 promoter. Data presented as mean ± SD; *p < 0.05 versus anti‐IgG, unpaired Student's *t*‐test. Western blot analysis of CDK6, ELF4‐Flag, and GAPDH expression in 293 T cells (G) or HEC‐1A cells (H) transfected with pCMV‐NCV or ELF4‐Flag vectors, using GAPDH as loading control. Band intensities quantified by ImageJ are mean ± SEM. **p < 0.01 versus pCMV‐NCV, unpaired Student's *t* test (G). *p < 0.05 versus pLVX‐puro, unpaired Student's *t*‐test (H).

The mRNA expression data from the TCGA UCEC cohort via GEPIA revealed a significant positive correlation (Figure 3A). With transfecting a pool of three ELF4 siRNAs into three EC cell lines, qRT‐PCR (Figure 3B) and western blot (Figure 3C,D) confirmed CDK6 reduction. To assess ELF4's transcriptional regulation of CDK6, we identified ELF4‐binding motifs in the CDK6 promoter (Figure S2A). Luciferase assays with truncated CDK6 promoter constructs pinpointed the −525 to +8 region as critical for activity (Figure S2B), with ELF4 overexpression in EC cells enhancing promoter activity (Figure 3E), particularly via motifs at −460 and −174. ChIP analysis in AN3CA and HEC‐1A cells confirmed ELF4 binding to these sites (Figure 3F). Additionally, ELF4 overexpression in 293 T cells (Figure 3G) and stable expression in HEC‐1A cells (Figure 3H) increased CDK6 levels. These data indicate that ELF4 transcriptionally upregulates CDK6, potentially driving EC progression.

### 3.4 Elevated CDK6 Expression Correlates With Clinicopathological Features and Poor Prognosis in EC

**Figure 4:**
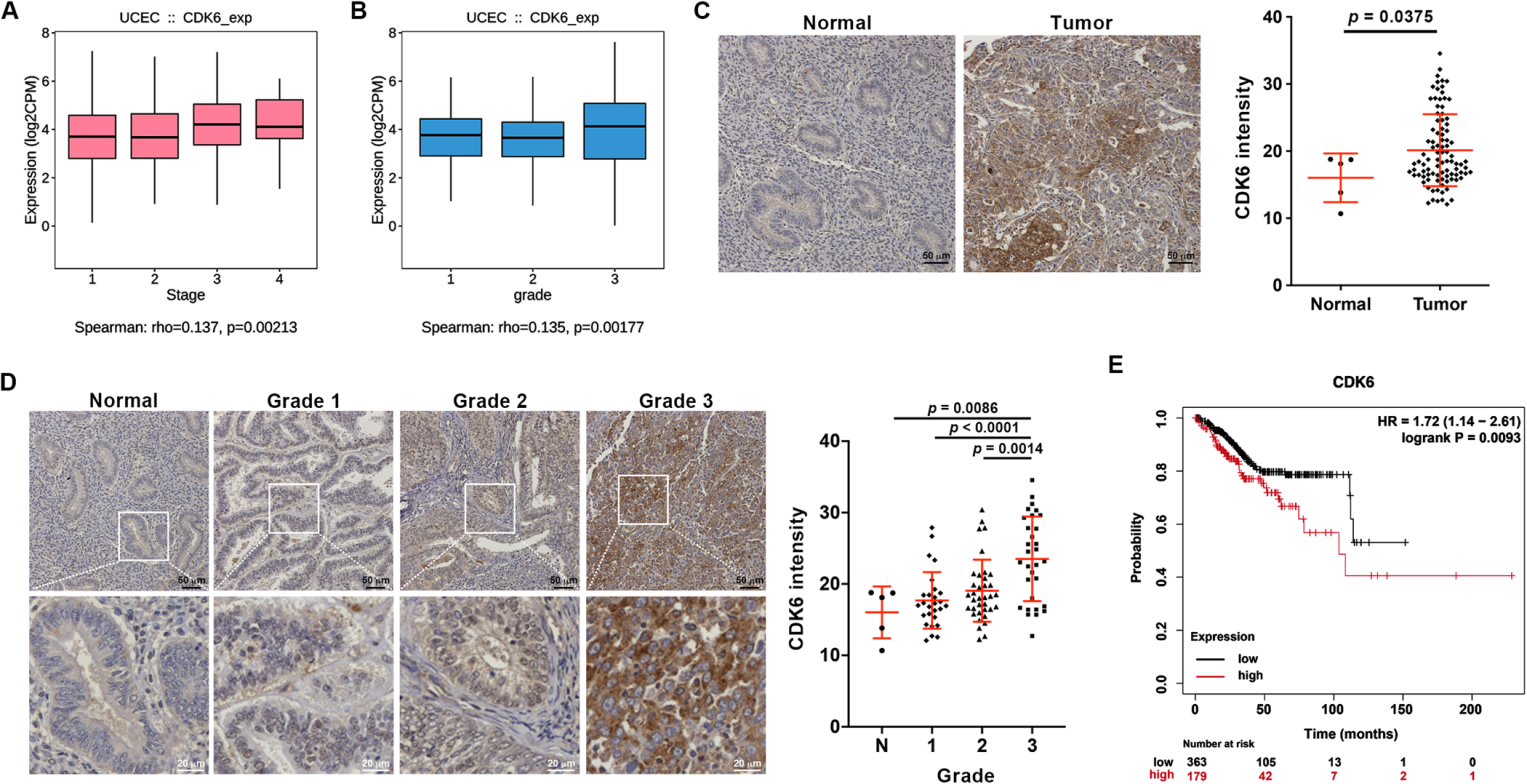
Upregulated CDK6 in EC contributes to poor prognosis. Spearman correlation analysis of CDK6 expression with pathological stages (A) or histological grades (B) in UCEC data from TCGA database, analyzed using TISIDB. (C) IHC analysis of CDK6 protein expression in normal and EC tissues on tissue microarray slides. Left panel: Representative images of CDK6 staining in normal uterine and EC tumor tissues. Right panel: Quantification of staining intensity, expressed as mean ± SD; *p* value was adjusted by Tukey's multiple comparison test. (D) Left panel: Representative images of CDK6 staining intensity across histological grades of EC tissues, with insets showing magnified view of indicated area. Right panel: Quantification of staining intensity, expressed as mean ± SD; *p* values were adjusted by Tukey's multiple comparison test. (E) Overall survival (OS) curves for UCEC patients in the TCGA database, stratified by CDK6 mRNA expression levels (upper tertile cutoff), generated using the Kaplan‐Meier plotter tool. Significance assessed by log‐rank test.

Given ELF4's regulation of CDK6, we evaluated CDK6's clinical relevance in EC. TISIDB analysis revealed strong associations between CDK6 expression and FIGO stage (Figure 4A) and tumor grade (Figure 4B). IHC analysis of an EC tissue microarray showed significantly higher CDK6 levels in EC specimens versus normal endometrial tissues (Figure 4C), with a positive correlation to tumor grade (Figure 4D). To evaluate prognostic impact, we performed Kaplan‐Meier analysis of OS using TCGA UCEC data, stratified by upper tertile CDK6 expression. Patients with elevated CDK6 exhibited significantly shorter OS than those with lower levels (Figure 4E). These findings link higher CDK6 expression to aggressive clinicopathological features and poor EC prognosis, suggesting its role in disease progression.

### 3.5 Both ELF4 and CDK6 Promote Self‐Renewal of EC‐CSCs

**Figure 5:**
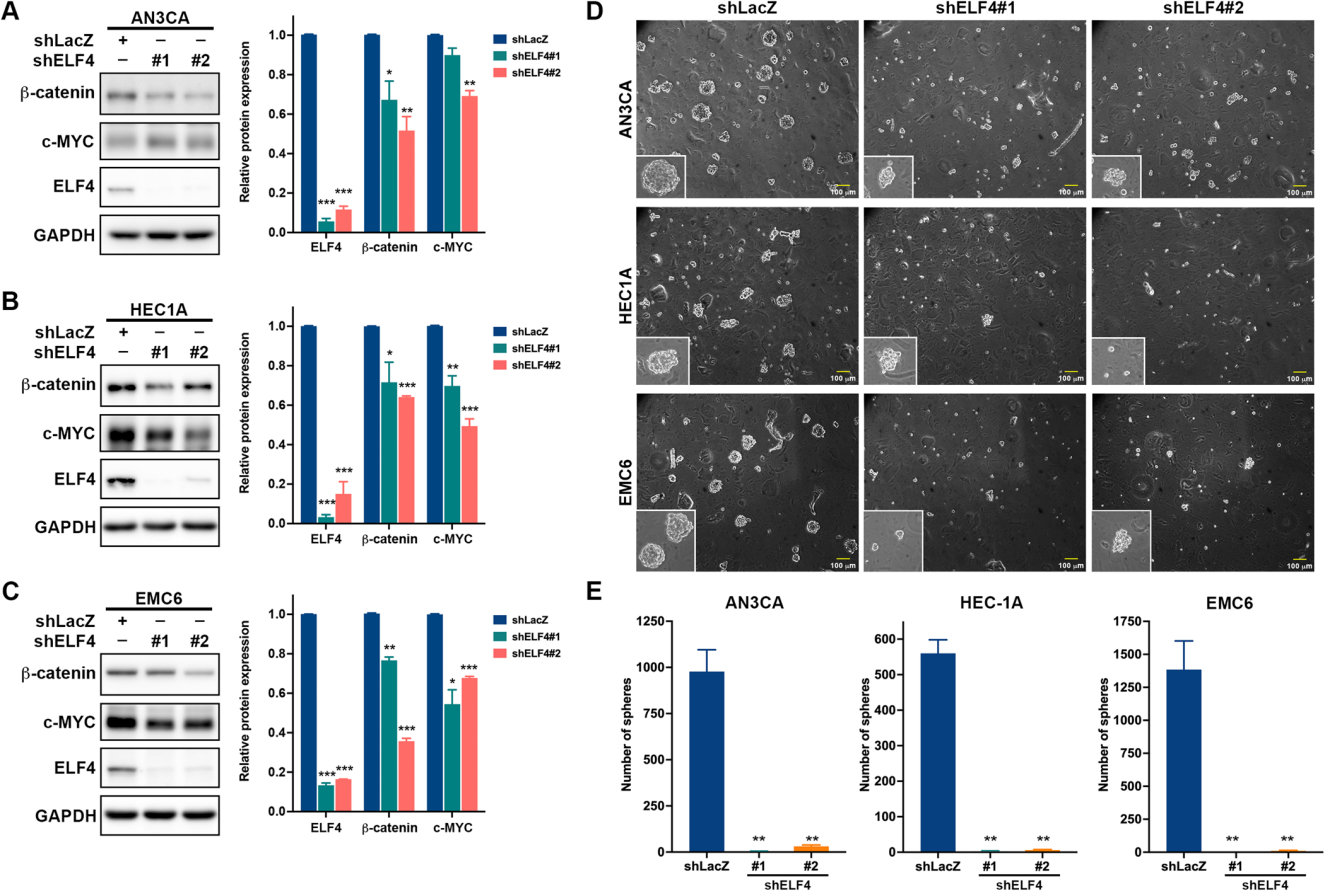
Knockdown of ELF4 reduces β‐catenin and c‐MYC protein levels and inhibits tumorsphere formation. Western blot analysis of β‐catenin, c‐MYC, and GAPDH expression in (A) AN3CA, (B) HEC‐1A, and (C) EMC6 cells transduced with shLacZ or ELF4‐specific short hairpin RNAs (shELF#1 or shELF4#2), using GAPDH as loading control. Band intensities are mean ± SEM; ***p < 0.001 versus shLacZ, unpaired Student's *t* test. Tumorsphere formation assay in cells transduced with shLacZ or ELF4‐specific shRNAs (shELF#1 or shELF4#2), assessed by (D) imaging and (E) counting tumorspheres after 7‐10 days using an inverted light microscope. Data are presented as mean ± SD; *p < 0.05; **p < 0.01; ***p < 0.001 versus shLacZ‐transduced cells, unpaired Student's *t*‐test.

**Figure 6:**
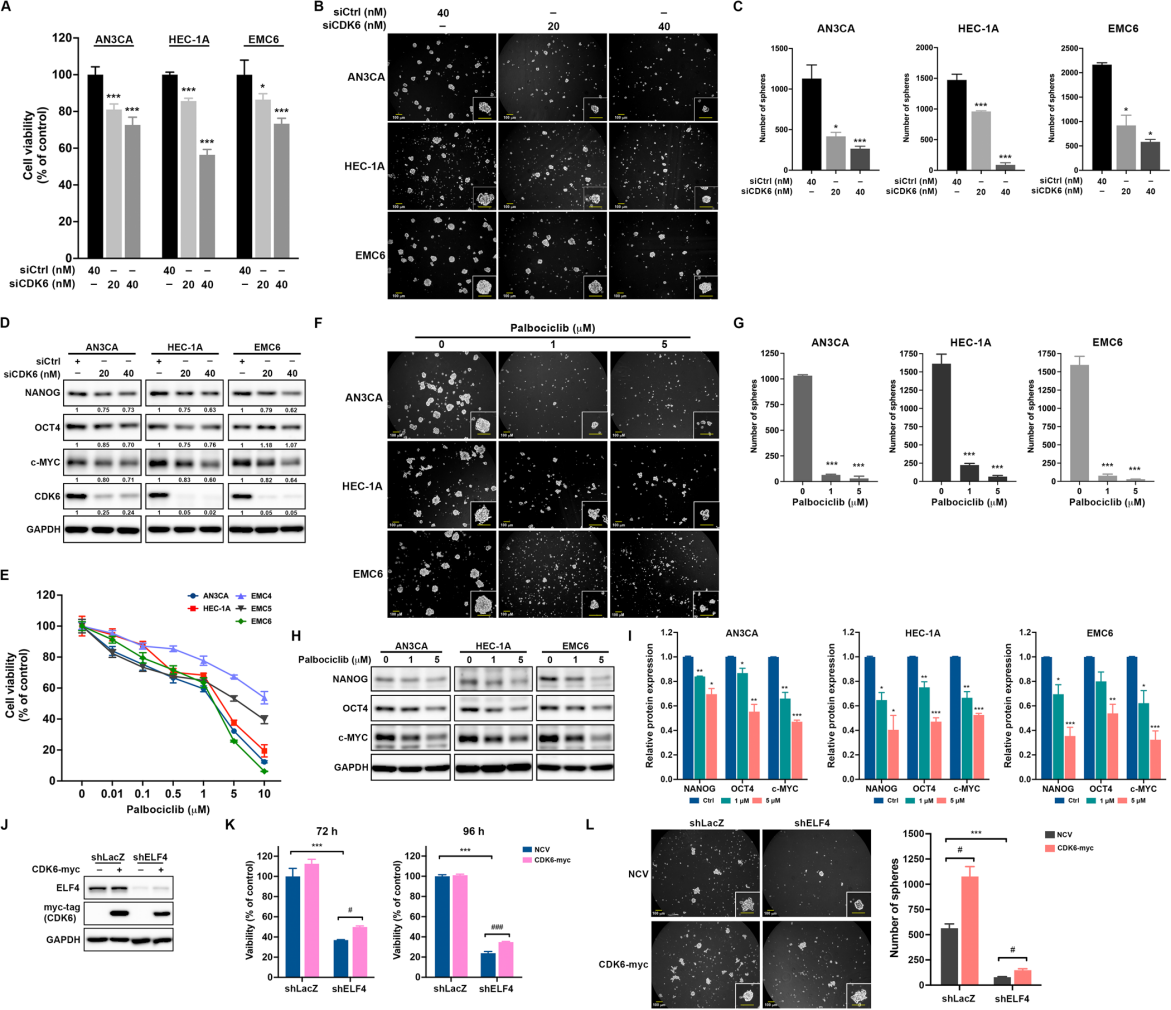
Inhibition of CDK6 blocks self‐renewal activity in EC. (A) MTT assay of cell viability in AN3CA, HEC‐1A, and EMC6 cells transfected with control (siCtrl) or CDK6‐specific small interfering RNAs (siCDK6), plated at 3000 cells/well in 96‐well plates. Data are mean ± SD; *p < 0.05; ***p < 0.001 versus siCtrl, unpaired Student's *t*‐test. Tumorsphere formation assay in AN3CA, HEC‐1A, and EMC6 cells transfected with siCtrl or siCDK6, assessed by (B) imaging and (C) counting tumorspheres after 7–10 days using an inverted light microscope. Data are presented as mean ± SD; *p < 0.05; ***p < 0.001 versus siCtrl, unpaired Student's *t*‐test. (D) Western blot analysis of CDK6, OCT4, NANOG, c‐MYC, and GAPDH expression in AN3CA, HEC‐1A, and EMC6 cells transfected with siCtrl or siCDK6, using GAPDH as loading control. Relative band intensities versus siCtrl were indicated. (E) MTT assay of cell viability in AN3CA, HEC‐1A, EMC4, EMC5, and EMC6 cells seeded at 3000 cells/well in 96‐well plates and treated with increasing Palbociclib doses. Data are presented as relative viability (mean ± SD) versus vehicle controls. Tumorsphere formation assay in AN3CA, HEC‐1A, and EMC6 cells seeded at 1 × 10⁴ cells/well in ultra‐low attachment 6‐well plates, treated with Palbociclib or vehicle for 7–10 days, assessed by (F) imaging and (G) counting tumorspheres using an inverted light microscope. Scale bar: 100 μm. Data presented as are mean ± SD; ***p < 0.001 versus vehicle, unpaired Student's *t*‐test. (H, I) Western blot analysis of NANOG, OCT4, c‐MYC, and GAPDH expression in AN3CA, HEC‐1A, and EMC6 cells treated with Palbociclib or vehicle, using GAPDH as loading control. Relative band intensities versus vehicle from three independent repeats were shown in (I). *p < 0.05; **p < 0.01; ***p < 0.001 vs vehicle control (Ctrl), unpaired Student's *t*‐test. (J, K, L) HEC‐1A cells were transduced with lentiviruses carrying shLacZ or shELF4 sequences followed by transfecting with vectors of empty (NCV) or myc‐tagged CDK6 cDNA (CDK6‐myc). The expressions of myc‐tagged CDK6 and ELF4 were confirmed by western blot (J). Cell viability at 72 h or 96 h post‐transduction was determined by MTT (K). **p < 0.01; ***p < 0.001; ^#^ *p* < 0.05; ^###^ *p* < 0.001. CSC activity was examined by tumorsphere assay (L).

ELF4 knockdown by shRNAs reduced protein levels of β‐catenin and c‐MYC, key CSC regulators (Fraszczak and Barczynski 2024), across all three cell lines (Figure 5A–C). Tumorsphere assays, enriching CSCs (Johnson et al. 2013; Lee et al. 2016), showed that ELF4 silencing decreased sphere size and number (Figure 5D, E). Likewise, CDK6 knockdown via siRNA, with efficiency validated (Figure S3), reduced viability (Figure 6A) and tumorsphere size and number (Figure 6B,C) in these cell lines, while lowering NANOG, OCT4, and c‐MYC expression (Figure 6D). Complementing gene silencing, Palbociclib, a CDK4/6 inhibitor, dose‐dependently reduced viability in AN3CA, HEC‐1A, and patient‐derived EMC4, EMC5, and EMC6 cells (Figure 6E), and decreased tumorsphere size and number (Figures 6F and 6G) and NANOG, OCT4, and c‐MYC levels (Figure 6H,I) in AN3CA, HEC‐1A, and EMC6 cells. Furthermore, overexpression of CDK6 in HEC‐1A cells (Figure 6J) partially restored cell viability following ELF4 knockdown at 72 h or 96 h timepoints (Figure 6K) and slightly reversed the impaired tumorsphere formation ability (Figure 6L). Taken together, these findings demonstrate that both ELF4 and CDK6 promote EC‐CSC self‐renewal.

### 3.6 TRIB3 Cooperates With ELF4 to Upregulate CDK6 Expression in EC

**Figure 7:**
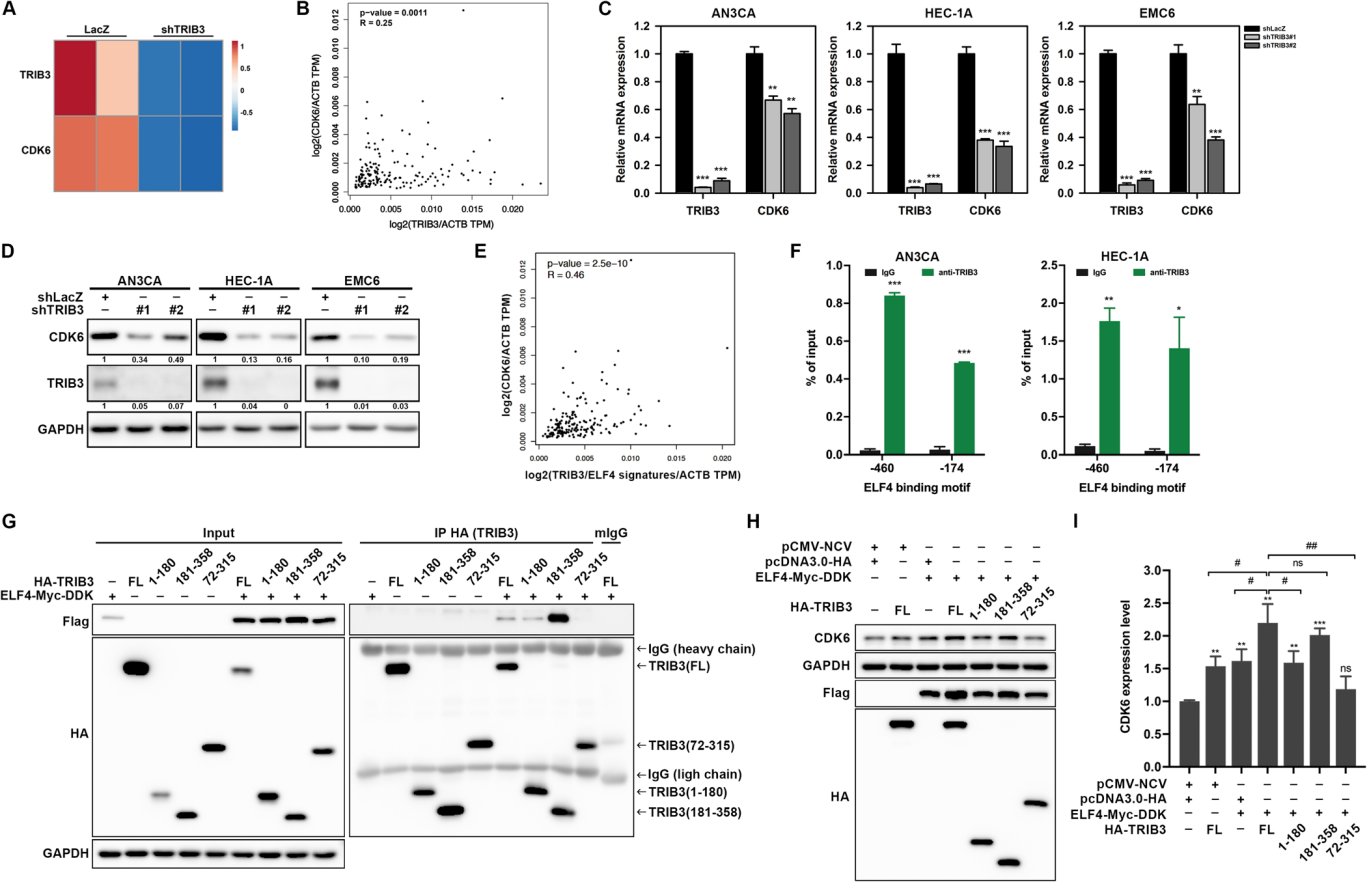
TRIB3 cooperates with ELF4 to regulate CDK6 expression in EC. (A) Heatmap of CDK6 expression in TRIB3‐knocked‐down HEC‐1A cells, generated using ClustVis from two independent replicates. (B) Spearman's correlation analysis of TRIB3 and CDK6 expression in UCEC patients of TCGA database, performed using GEPIA2. (C) Relative mRNA levels of TRIB3 and CDK6 in AN3CA, HEC‐1A, and EMC6 cells transduced with shLacZ or TRIB3‐specific short hairpin RNAs (shTRIB3#1 or shTRIB3#2), measured by SYBR‐Green‐based qRT‐PCR. Data are presented as fold changes (mean ± SD) versus shLacZ; **p < 0.01; ***p < 0.001. (D) Western blot analysis of CDK6, TRIB3, and GAPDH expression in AN3CA, HEC‐1A, and EMC6 cells transduced with shLacZ or TRIB3‐specific shRNAs, using GAPDH as loading control. Relative band intensities versus shLacZ were indicated. (E) Spearman's correlation analysis of TRIB3/ELF4 and CDK6 expression in UCEC patients of TCGA database, performed using GEPIA2. (F) ChIP‐qPCR assay assessing TRIB3 binding to putative ELF4 binding elements on the CDK6 promoter in AN3CA and HEC‐1A cells, using anti‐TRIB3 antibody or normal rabbit IgG as control. Data are presented as mean ± SD; *p < 0.05; **p < 0.01, ***p < 0.001 versus anti‐IgG, unpaired Student's *t*‐test. (G) 293 T cells were transfected with the indicated plasmids for 48 h and total cell lysates were extracted as input. After IP with anti‐HA or control mouse IgG (mIG) antibodies, the pull down proteins were subjected to western blot for HA tagged TRIB3 fragments or flag tagged ELF4. (H, I) 293 T cells were transfected with indicated plasmids for 48 h and total cell lysates were extracted and subjected to western blot for analyzing the levels of flag tagged ELF4, HA tagged TRIB3 fragments, and CDK6 (H). GAPDH was used as a protein loading control. The quantitative data of band intensities were quantified by ImageJ three independent experiment repeats are presented as mean ± SEM (I). **p < 0.01; ***p < 0.001; ^#^ *p* < 0.05; ^##^ *p* < 0.01 by unpaired Student's *t*‐test. n.s., not significant.

We previously demonstrated that TRIB3 interacts with ELF4 to regulate β‐catenin transcription (Wang et al. 2020). To further explore this interaction, the ELF4 and TRIB3 protein sequences were submitted to AlphaFold (Abramson et al. 2024) with the objective of generating a combined structural model (Figure S4). Five putative interaction models were obtained, with model_0 and model_1 showing identical highest‐ranking scores (0.89) (Table S3). Model_1 was selected for further analysis due to its slightly lower fraction disordered value (0.64 vs 0.66) and lower chain_pair_pae_min value (3.0 vs 3.33) (Table S3). The selected model was further analyzed using ClusPro (Jones et al. 2022) to predict interaction regions (Table S4) and PRODIGY (Xue et al. 2016) to determine the dissociation constant (Table S5) and key interacting residues (Table S6). The PRODIGY analysis revealed that TRIB3 and ELF4 interact primarily through TRIB3's pseudokinase domain (aa 131–314) and ELF4's ETS domain (aa 207–291) (Table S6). A reanalysis of RNA‐seq data from TRIB3‐knockdown HEC‐1A cells, as previously reported (Wang et al. 2020), revealed a reduction in CDK6 expression (Figure 7A). GEPIA analysis of UCEC patients from the TCGA database showed a positive correlation between TRIB3 and CDK6 expression (Figure 7B). Consistently, TRIB3 knockdown in AN3CA, HEC‐1A, and EMC6 cells decreased CDK6 mRNA (Figure 7E) and protein (Figure 7D) levels. GEPIA analysis further indicated a significant correlation among CDK6, TRIB3, and ELF4 expression in UCEC patients (Figure 7E). ChIP assays confirmed TRIB3 enrichment at ELF4‐binding motifs (‐460 and ‐174 bp) within the CDK6 promoter (Figure 7F). To experimentally validate and map these domains, we constructed HA‐tagged TRIB3 fragments (amino acids 1–180, 181–358, and 72–315) along with full‐length TRIB3 (amino acids 1–358) and co‐transfected each separately with full‐length Myc‐DDK‐tagged ELF4 into 293 T cells, followed by anti‐HA co‐immunoprecipitation (Co‐IP) analysis (Figure 7G). Consistent with the computational predictions, the results revealed no detectable interaction between the TRIB3 72–315 fragment (which overlaps most of the pseudokinase domain but may lack critical flanking residues) and ELF4, an interaction with the 1–180 fragment (encompassing the N‐terminal portion of the pseudokinase domain) comparable to that of full‐length TRIB3, and the strongest interaction with the 181–358 fragment, even stronger than full‐length TRIB3 (Figure 7G). We next assessed CDK6 protein levels by immunoblotting whole‐cell lysates from 293 T cells co‐transfected with these TRIB3 fragments and full‐length ELF4, observing a marked increase in CDK6 expression with the full length (2.2‐fold vs. empty vector control), 181–358 fragment (2.1‐fold), a moderate increase with the 1–180 fragment (1.6‐fold), and no change with the 72–315 fragment (Figures 7H and 7I). Taken together, these findings support the role of TRIB3's pseudokinase domain, particularly its C‐terminal portion (amino acids 181–314), in mediating ELF4 interaction and downstream CDK6 induction in EC cells.

### 3.7 The Combination of CDK6 and β‐Catenin Inhibition Exerts Stronger Suppression of EC‐CSC Activity

**Figure 8:**
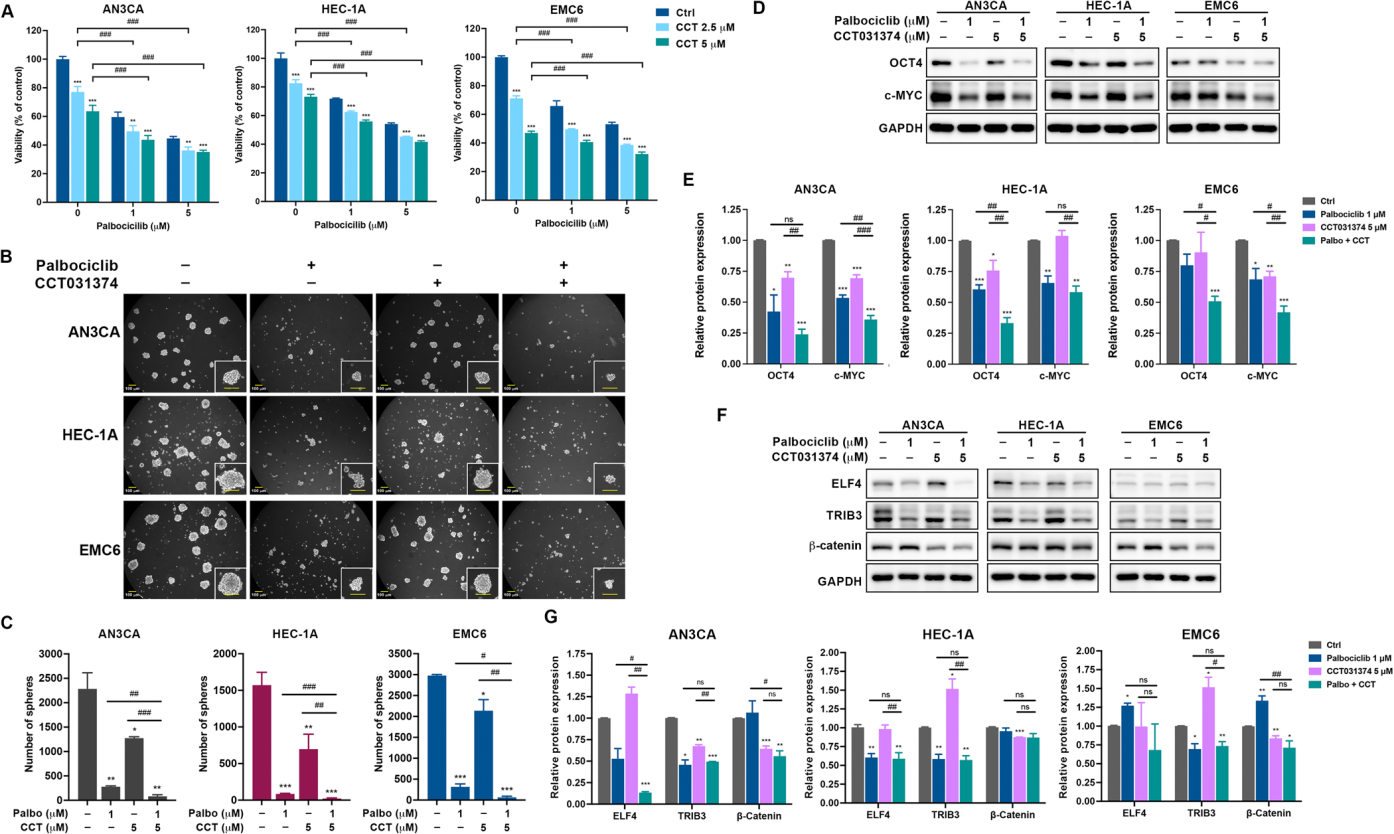
Combination of CDK6 and β‐catenin inhibition enhances EC‐CSC targeting effects. (A) Cells were treated with palbociclib and/or CCT031374 (CCT) as indicated. After 72 h, cell viability was measured by MTT assay. **p < 0.01; ***p < 0.001, compared to cells without CCT treatment. ^###^ *p* < 0.001. Cells were treated with 5 μM palbociclib, 5 μM CCT, or a combination of both drugs at 5 μM, followed by tumorsphere cultivation. Tumorspheres were imaged and counted using inverted microscopy on Day 7 (B); quantification data are shown in (C). *p < 0.05; **p < 0.01; ***p < 0.001, compared to nontreated cells. ^#^ *p* < 0.05; ^##^ *p* < 0.01; ^###^ *p* < 0.001. (D, E, F, G) Cells were treated with 5 μM palbociclib, 5 μM CCT, or both drugs in combination for 48 h. Protein expression levels of OCT4 and c‐MYC (D, E), or ELF4, TRIB3, and β‐catenin (F, G), were analyzed by western blot. Quantitative data of band intensities in (E) and (G) resulted from three independent experimental repeats are quantified by ImageJ and are presented as mean ± SEM. represent results. *p < 0.05; **p < 0.01; ***p < 0.001, compared to non‐treated cells. ^#^ *p* < 0.05; ^##^ *p* < 0.01; ^###^ *p* < 0.001. n.s., not significant.

Building on our previous demonstration that TRIB3 positively regulates β‐catenin expression and contributes to EC‐CSC maintenance through its interaction with ELF4 (Wang et al. 2020), we hypothesized that combined inhibition of CDK6 and β‐catenin would synergistically suppress EC‐CSCs. To test this, we assessed cell viability in three EC cell lines (AN3CA, HEC‐1A, and EMC6) treated with the CDK6 inhibitor palbociclib and the β‐catenin inhibitor CCT031374 (Ewan et al. 2010). The combination treatment at 5 µM each resulted in the lowest viability across all cell lines (Figure 8A). Tumorsphere formation assays further demonstrated that the combined treatment induced the strongest suppression of sphere‐forming capability (Figure 8B,C). Consistent with this, immunoblotting revealed the most pronounced decreases in cancer stemness markers c‐Myc and OCT4 under combination treatment (Figure 8D,E). Moreover, evaluation of ELF4, TRIB3, and β‐catenin protein levels showed significant suppression of all three proteins with co‐treatment of palbociclib and CCT031374 (Figure 8F,G). Collectively, these data indicate that the TRIB3/ELF4‐mediated upregulation of CDK6 and β‐catenin plays a key role in maintaining EC‐CSC activity, and their combined inhibition offers enhanced therapeutic potential.

### 3.8 Co‐Expression of CDK6, ELF4, and TRIB3 Predicts Poor Prognosis in EC

**Figure 9:**
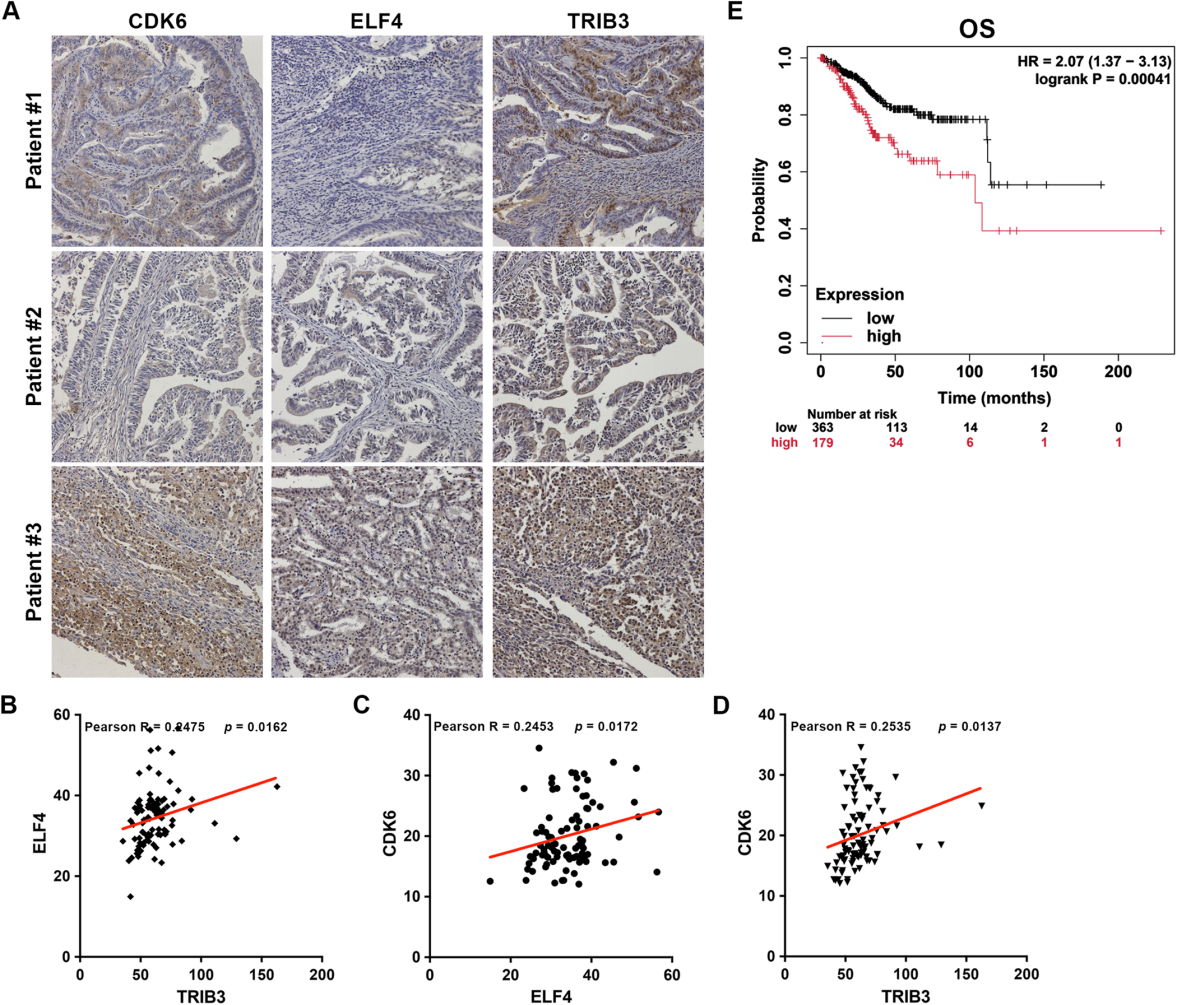
ELF4 and CDK6 expressions show a positive correlation in EC specimens. (A) Representative IHC images of ELF4, TRIB3, and CDK6 staining intensities in slides of an EC tissue microarray. (B) Pearson's correlation analysis of ELF4 and TRIB3 expressions in EC specimens. (C) Pearson's correlation analysis of ELF4 and CDK6 expression in EC specimens. (D) Pearson's correlation analysis of TRIB3 and CDK6 expression in EC specimens. (E) Overall survival (OS) curves for UCEC patients in the TCGA database, stratified by co‐expression of ELF4, TRIB3, and CDK6 mRNA (upper tertile cutoff), generated using the Kaplan‐Meier plotter tool. Significance assessed by log‐rank test.

Building on our evidence that ELF4 and TRIB3 upregulate CDK6 in EC cells, we evaluated protein levels of ELF4, TRIB3, and CDK6 in a tissue microarray of 97 EC specimens by IHC (Figure 9A). Expression analysis showed a positive correlation between ELF4 and TRIB3 (Figure 9B), with CDK6 strongly associated with both ELF4 (Figure 9C) and TRIB3 (Figure 9D). Using TCGA UCEC data, we performed Kaplan‐Meier analysis of OS based on upper tertile values of combined ELF4/TRIB3/CDK6 gene expression. Patients with high co‐expression exhibited the shortest OS (Figure 9E). These findings reinforce CDK6 as a downstream effector of the ELF4/TRIB3 transcriptional complex, driving EC progression, and indicate that their co‐expression robustly predicts poor prognosis in EC.

## 4 Discussion

While abnormal ETS transcription factor expression influences biological processes in various malignant tumors (Suico et al. 2017), the regulatory mechanisms and clinical significance of ELF4 in EC remain poorly understood. Mounting evidence suggests ELF4 drives tumorigenesis and cancer progression through diverse mechanisms. In glioblastoma, high ELF4 expression promotes proliferation and stemness, reducing patient survival (Bazzoli et al. 2012). Similarly, in clear cell renal cell carcinoma (ccRCC), elevated ELF4 levels correlate with worse outcomes, enhancing proliferation, migration, invasion, M2 macrophage polarization, and chemotaxis toward RCC cells (Lu et al. 2023). Additionally, Kafita et al. reported that frequent ELF4 amplification across cancers is associated with poor prognosis and increased drug resistance (Kafita et al. 2021). In a mouse model, Elf4 (Mef) knockout in p53‐deficient mice delayed lymphoma onset compared to p53 knockout alone, likely due to p16 tumor suppressor activation (Sashida et al. 2009). Recent research shows FGF19 induces ELF4 in colorectal cancer, driving metastasis via ELF4‐mediated FGFR4 and Src kinase signaling (Chen et al. 2023). Notably, FP‐1039, a soluble FGFR1 Fc fusion protein, inhibits growth in HEC‐1B EC cells (Harding et al. 2013), suggesting a potential link to FGFR signaling. Whether FGFR signaling induces ELF4 in EC merits further exploration.

Hua et al. (2019) demonstrated a positive feedback loop between TRIB3 and β‐catenin in colorectal cancer (CRC) cells. Specifically, knockdown of TRIB3 reduced TOPFlash reporter activity, indicating diminished Wnt/β‐catenin signaling. Furthermore, activation of β‐catenin by Wnt3a increased TRIB3 expression, whereas knockdown of β‐catenin resulted in decreased TRIB3 protein levels in CRC cells (Hua et al. 2019). However, in our study, pharmacological inhibition of β‐catenin using CCT031374 did not reduce TRIB3 expression in HEC‐1A or EMC6 EC cells (see Figure 8F, G). These findings, together with our previous results (Wang et al. 2020), indicate that CDK6 and β‐catenin are downstream targets of the TRIB3/ELF4 signaling axis in EC cells, rather than participants in a reciprocal regulatory mechanism.

Beyond its established role in cell cycle regulation, CDK6 contributes to diverse biological and pathological processes, including angiogenesis (Kollmann et al. 2019), stem cell activation (Liu et al. 2020), immune response (Nebenfuehr et al. 2020), metastasis (Zhang et al. 2020), and drug resistance (Zhang et al. 2022). For instance, Kollmann et al. demonstrated that CDK6 participates in a transcriptional complex that positively regulates VEGF‐A expression in a mouse model of B‐acute lymphoid leukemia, thereby promoting tumor angiogenesis (Kollmann et al. 2013). Similarly, in leukemic stem cells, CDK6 knockout prevented disease induction in transplantation models, an effect mediated by its transcriptional regulation of the early growth response 1 (Egr1) gene (Scheicher et al. 2015). In our study, treatment with Palbociclib, a CDK4/6 inhibitor, significantly suppressed both cell proliferation and tumorsphere formation in endometrial cancer (EC) cells (Figure 6). Notably, we found that both CDK6 knockdown and Palbociclib treatment markedly reduced the expression of stemness markers OCT4 and NANOG (Figure 6). These results highlight CDK6's critical roles in driving EC cell proliferation and sustaining cancer stem cell‐like properties. This dual impact of CDK6 inhibition suggests its potential as a target for more comprehensive and effective therapeutic strategies.

TRIB3, a pseudokinase lacking typical enzymatic activity, functions as a scaffold to modulate the activities of interacting proteins, including transcription factors (TFs). For instance, Li et al. demonstrated that TRIB3 binds MYC, protecting it from UBE3B‐mediated degradation, a process linked to lymphoma cell proliferation and self‐renewal (Li et al. 2020). Disrupting this TRIB3‐MYC interaction reduced tumor burden in a mouse lymphoma model and patient‐derived xenografts (Li et al. 2020). In contrast, TRIB3 can repress certain TFs, as seen in liver cancer cells treated with bortezomib, where it binds to ATF4 on chromatin (Ord et al. 2021). This enrichment at ATF4‐bound regions suppresses C/EBP–ATF‐driven transcription of ER stress response genes, potentially contributing to bortezomib resistance (Ord et al. 2021). Interestingly, our study reveals that TRIB3 can also enhance TF activity, as co‐expression of TRIB3 with ELF4 increased ELF4‐driven CDK6 expression (Figure 7G). Collectively, these findings indicate that TRIB3's effects on TFs are highly context‐dependent, ranging from enhancement to repression depending on the specific TF and cellular environment.

## 5 Conclusion

In conclusion, the present study demonstrates that elevated expression of ELF4 is significantly associated with higher histological grading and adverse patient prognosis in EC. Inhibition of ELF4 expression has been shown to effectively reduce cell growth and self‐renewal capability of EC cells by attenuating the expression of CDK6 and β‐catenin/c‐MYC. TRIB3 has also been identified as a crucial factor for cooperation with ELF4 to upregulate CDK6 expression. The suppression of CDK6, achieved either through genetic knockdown or pharmacological inhibition with Palbociclib, has been shown to significantly curtail EC‐CSC properties, including tumorsphere formation and expression of stemness markers. The present study has elucidated a novel mechanism wherein ELF4/TRIB3 regulates CDK6 expression, which is responsible for maintaining stemness in ECs. This ELF4‐TRIB3‐CDK6 axis is identified as a promising therapeutic target for EC treatment by suppressing both tumor growth and CSC population.

## Author Contributions

Conceptualization: Wen‐Ling Wang, and Wen‐Wei Chang. Data curation and analysis: Chun‐Yu Chen, Yueh‐Chun Lee, Yu‐Hao Huang, and Wen‐Ling Wang. Funding acquisition: Chun‐Yu Chen and Wen‐Wei Chang. Original draft writing: Yueh‐Chun Lee and Wen‐Ling Wang. Manuscript review and editing: Chun‐Yu Chen, Yueh‐Chun Lee, and Wen‐Wei Chang. All authors have read and agreed to the published version of the manuscript.

## Ethics Statement

This study utilized a human endometrial cancer tissue microarray (TMA; Cat. No. EM1021c) obtained from TissueArray.Com LLC. The use of this tissue microarray was reviewed and approved by the Institutional Review Board (IRB) of Tungs' Taichung MetroHarbor Hospital (Taichung City, Taiwan) with Approval No. 114022.

## Consent

As the study involved only commercially acquired, de‐identified tissue samples and no direct interaction with human participants, the requirement for informed consent was waived by the IRB. All procedures were conducted in accordance with ethical standards for the treatment of human research materials.

## Conflicts of Interest

The authors declare no conflicts of interest.

## Supporting information


**Figure S1:** Knockdown efficiency of ELF4 shRNA in EC cells. AN3CA, HEC‐1A and EMC6 cells were transduced with lentiviruses carrying shLacZ or ELF4‐specific shRNAs and selected with 2 µg/ml puromycin for three days. **Figure S2:** Human CDK6 promoter activity in AN3CA and EMC6 cells. (A) 5’ deletion constructs of the CDK6 promoter. The potential ELF4 regulatory regions were indicated as red bars. (B) AN3CA and EMC6 cells were transfected with pGL3‐luciferase reporter plasmids containing CDK6 promoter constructs or pGL3‐basic luciferase reporter for 48 h. **Figure S3:** The knockdown efficiency of CDK6 siRNA in EC cells. AN3CA (A), HEC‐1A (B) and EMC6 (C) cells were transfected with 40 nM of negative control siRNA (siCtrl), or 20 and 40 nM CDK6 siRNAs (siCDK6) for 24 hours using TransIT‐X2 transfection reagent. The protein levels of CDK6 were determined by western blot analysis. **Figure S4:** Predicted interaction between ELF4 and TRIB3. (A) and (B) depict the full‐length protein structures of ELF4 and TRIB3, respectively, as predicted by AlphaFold. (C) illustrates a docking model of the predicted complex, with the TRIB3 interaction region highlighted in dark blue and the ELF4 interaction region in dark pink, against lighter shades for the full structures. Interaction regions were predicted based on high‐confidence binding scores from AlphaFold. **Table S1:** Primer sequences used in this study. **Table S2:** Antibodies used in this study. **Table S3:** The predicted confidences of ELF4 and TRIB3 interaction by AlphaFold. **Table S4:** The predicted molecular docking between ELF and TRIB3a. **Table S5:** Binding affinity and Kd prediction between ELF4 and TRIB3a. **Table S6:** The interacting residues between ELF4 and TRIB3a.

IRB 114022.

western blot‐raw results_1031.

## Data Availability

The data supporting this study are available from the corresponding author upon reasonable request.
