# Supplementary material for: ELF4/TRIB3/CDK6 Axis Promotes Cancer Stem Cell Activity in Endometrial Cancer
Source: J Cell Physiol. 2025 Nov 25;240(11):e70113. doi: 10.1002/jcp.70113 (PMC12645360; doi:10.1002/jcp.70113)
Supplement: Supplementary file 1 — Figure S1: Knockdown efficiency of ELF4 shRNA in EC cells. AN3CA, HEC‐1A and EMC6 cells were transduced with lentiviruses carrying shLacZ or ELF4‐specific shRNAs and selected with 2 µg/ml puromycin for three days. Figure S2: Human CDK6 promoter activity in AN3CA and EMC6 cells. (A) 5’ deletion constructs of the CDK6 promoter. The potential ELF4 regulatory regions were indicated as red bars. (B) AN3CA and EMC6 cells were transfected with pGL3‐luciferase reporter plasmids containing CDK6 promoter constructs or pGL3‐basic luciferase reporter for 48 h. Figure S3: The knockdown efficiency of CDK6 siRNA in EC cells. AN3CA (A), HEC‐1A (B) and EMC6 (C) cells were transfected with 40 nM of negative control siRNA (siCtrl), or 20 and 40 nM CDK6 siRNAs (siCDK6) for 24 hours using TransIT‐X2 transfection reagent. The protein levels of CDK6 were determined by western blot analysis. Figure S4: Predicted interaction between ELF4 and TRIB3. (A) and (B) depict the full‐length protein structures of ELF4 and TRIB3, respectively, as predicted by AlphaFold. (C) illustrates a docking model of the predicted complex, with the TRIB3 interaction region highlighted in dark blue and the ELF4 interaction region in dark pink, against lighter shades for the full structures. Interaction regions were predicted based on high‐confidence binding scores from AlphaFold. Table S1: Primer sequences used in this study. Table S2: Antibodies used in this study. Table S3: The predicted confidences of ELF4 and TRIB3 interaction by AlphaFold. Table S4: The predicted molecular docking between ELF and TRIB3a. Table S5: Binding affinity and Kd prediction between ELF4 and TRIB3a. Table S6: The interacting residues between ELF4 and TRIB3a. [file JCP-240-0-s002.docx]

**Supporting information**

**Methods**

**Prediction of interaction between ELF4 and TRIB3**

The 3D structure models of the ELF4-TRIB3 interaction were generated using the AlphaFold server (https://deepmind.google/technologies/alphafold/), yielding five putative interaction models. The 3D structures of ELF4 and TRIB3 were then extracted from model_1, which displayed the highest confidence, using PyMOL. Protein-protein binding affinity and complex formation were assessed using the ClusPro web server (https://cluspro.org/login.php?redir=/home.php), resulting in 10 putative ELF4-TRIB3 complex models. Subsequently, these 10 models were submitted to the PRODIGY web server (https://rascar.science.uu.nl/prodigy/) to calculate interface properties and binding energy (kcal/mol) and to predict interacting residues.

**Tables**

| **Table S1. Primer sequences used in this study** | |
| --- | --- |
| **Primer Name** | **Primer sequences** |
| **Primer for real-time PCR** |  |
| ELF4 sense: | 5'-AATTGGGACCGTCGCTAGACGA-3' |
| ELF4 antisense: | 5'-GTGGATGTTGCTGGGCACTGAA-3' |
| CDK6 sense | 5'-GGATAAAGTTCCAGAGCCTGGAG-3' |
| CDK6 antisense | 5'-GCGATGCACTACTCGGTGTGAA-3' |
| TRIB3 sense | 5'-ACCGTATCCCTGAGCCTGA-3' |
| TRIB3 antisense | 5'-CTTGTCCCACAGGGAATCAT-3' |
| GAPDH sense | 5'-CAATGACCCCTTCATTGACC-3' |
| GAPDH antisense | 5'-TGGACTCCACGACGTACTCA-3' |
|  |  |
| **Primers used in ChIP in the CDK6 promoter** |  |
| ELF4 binding site -174 sense | 5'-CTTGTGCGCGACCCCTGAAC-3' |
| ELF4 binding site -174 antisense | 5'-GGCACGTCAATGTCACGGCT-3' |
| ELF4 binding site -460 sense | 5'-TCTTCCCAAGGTTTCCACCAG-3' |
| ELF4 binding site -460 antisense | 5'-GGGTAAAGGAGTCTCGGTTG-3' |

| **Table S2. Antibodies used in this study** | | |
| --- | --- | --- |
| **Product** | **Source** | **No. of Catalogue** |
| **Primary antibody** |  |  |
| Western blot: |  |  |
| anti-CDK6 | Proteintech group Inc. | 14052-1-AP |
| anti-ELF4 | Santa Cruz Biotechnology, Inc | sc-515363 |
| anti-b-catenin | BD Biosciences | BD 610154 |
| anti-c-MYC | Proteintech group Inc. | 10828-1-AP |
| anti-OCT4 | Proteintech group Inc. | 11263-1-AP |
| anti-NANOG | ABclonal Inc. | A3232 |
| anti-TRIB3 | Proteintech group Inc. | 13300-1-AP |
| anti-GAPDH | GeneTex International Corporation | GTX100118 |
|  |  |  |
| **Co-Immunoprecipitation:** |  |  |
| anti-HA tag | Santa Cruz Biotechnology, Inc | sc-7392 |
| anti-Flag M2 | Sigma-Aldrich Corporation | F-1804 |
| normal mouse-IgG | Leadgene Biomedical Inc. | LDG0001YD |
|  |  |  |
| **Immunohistochemistry:** |  |  |
| anti-CDK6 | Proteintech group Inc. | 14052-1-AP |
| anti-ELF4 | Santa Cruz Biotechnology, Inc | sc-515363 |
| anti-TRIB3 | Proteintech group Inc. | 13300-1-AP |
|  |  |  |
| **Chromatin Immunoprecipitation:** | |  |
| anti-ELF4 | Santa Cruz Biotechnology, Inc | sc-515363 |
| anti-TRIB3 | Proteintech group Inc. | 13300-1-AP |
|  |  |  |
| **Secondary antibodies:** |  |  |
| anti-rabbit IgG-HRP | GeneTex International Corporation | GTX21311201 |
| anti-mouse IgG-HRP | GeneTex International Corporation | GTX213111-01 |
| EasyBlot anti mouse IgG-HRP | GeneTex International Corporation | GTX221667-01 |

|  | **chain_iptm** | **chain_pair_iptm** | **chain_pair_pae_min** | **chain_ptm** | **fraction disordered** | **has_clash** | **iptm** | **num_recycles** | **ptm** | **ranking_score** |
| --- | --- | --- | --- | --- | --- | --- | --- | --- | --- | --- |
| **model_0** | 0.62, 0.62 | 0.71, 0.62 | 0.76, 3.33 | 0.71, 0.22 | 0.66 | 0 | 0.62 | 10 | 0.36 | 0.89 |
|  |  | 0.62, 0.22 | 2.8, 0.76 |  |  |  |  |  |  |  |
| **model_1** | 0.62, 0.62 | 0.71, 0.62 | 0.76, 3.0 | 0.71, 0.22 | 0.64 | 0 | 0.62 | 10 | 0.36 | 0.89 |
|  |  | 0.62, 0.22 | 2.59, 0.76 |  |  |  |  |  |  |  |
| **model_2** | 0.59, 0.59 | 0.68, 0.59 | 0.76, 3.11 | 0.68, 0.22 | 0.66 | 0 | 0.59 | 10 | 0.35 | 0.87 |
|  |  | 0.59, 0.22 | 2.72, 0.76 |  |  |  |  |  |  |  |
| **model_3** | 0.58, 0.58 | 0.68, 0.58 | 0.76, 3.25 | 0.68, 0.23 | 0.66 | 0 | 0.58 | 10 | 0.36 | 0.87 |
|  |  | 0.58, 0.23 | 2.74, 0.76 |  |  |  |  |  |  |  |
| **model_4** | 0.59, 0.59 | 0.68, 0.59 | 0.76, 2.83 | 0.68, 0.23 | 0.64 | 0 | 0.59 | 10 | 0.34 | 0.86 |
|  |  | 0.59, 0.23 | 2.62, 0.76 |  |  |  |  |  |  |  |

**Table S3. The predicted confidences of ELF4 and TRIB3 interaction by AlphaFold**

**Table S4. The predicted molecular docking between ELF and TRIB3^a^**.

| **Cluster** | **Members** | **Representative** | **Weighted Score** |
| --- | --- | --- | --- |
| **0** | 74 | Center | -1684.3 |
| **0** | 74 | Lowest Energy | -2284.4 |
| **1** | 57 | Center | -1485.3 |
| **1** | 57 | Lowest Energy | -2123.3 |
| **2** | 46 | Center | -1354.6 |
| **2** | 46 | Lowest Energy | -1575 |
| **3** | 40 | Center | -1427 |
| **3** | 40 | Lowest Energy | -1556.6 |
| **4** | 31 | Center | -1610.8 |
| **4** | 31 | Lowest Energy | -1649.1 |
| **5** | 26 | Center | -1364.6 |
| **5** | 26 | Lowest Energy | -1457.9 |
| **6** | 24 | Center | -1338.7 |
| **6** | 24 | Lowest Energy | -1484.8 |
| **7** | 21 | Center | -1222.3 |
| **7** | 21 | Lowest Energy | -1465.7 |
| **8** | 19 | Center | -1291 |
| **8** | 19 | Lowest Energy | -1442.5 |
| **9** | 18 | Center | -1298.4 |
| **9** | 18 | Lowest Energy | -1340.1 |
| **10** | 17 | Center | -1594.6 |
| **10** | 17 | Lowest Energy | -1594.6 |

^a^The predictions were done by ClusPro server (https://cluspro.org/).

**Table S5. Binding affinity and Kd prediction between ELF4 and TRIB3^a^.**

| **Protein-protein complex** | **ΔG (kcal mol-1)** | **Kd (M) at ℃** | **ICs charged-charged** | **ICs charged-polar** | **ICs charged-apolar** | **ICs polar-polar** | **ICs polar-apolar** | **ICs apolar-apolar** | **NIS charged** | **NIS apolar** |  |
| --- | --- | --- | --- | --- | --- | --- | --- | --- | --- | --- | --- |
| model_000_00 | -23.2 | 9.00E-18 | 24 | 16 | 68 | 0 | 45 | 70 | 23.09 | 47.07 |  |
| model_000_01 | -23.4 | 7.40E-18 | 22 | 14 | 75 | 1 | 44 | 70 | 22.64 | 47.25 |  |
| model_000_02 | -17.8 | 8.10E-14 | 20 | 21 | 54 | 3 | 32 | 42 | 22.53 | 47.91 |  |
| model_000_03 | -16.2 | 1.20E-12 | 14 | 20 | 53 | 6 | 30 | 51 | 22.79 | 47.22 |  |
| model_000_04 | -19 | 1.10E-14 | 7 | 6 | 43 | 4 | 48 | 66 | 22.89 | 47.1 |  |
| model_000_05 | -17 | 3.60E-13 | 13 | 20 | 62 | 3 | 27 | 52 | 22.45 | 47.37 |  |
| model_000_06 | -15.2 | 6.70E-12 | 18 | 19 | 43 | 2 | 25 | 54 | 22.52 | 47.55 |  |
| model_000_07 | -15.9 | 2.10E-12 | 23 | 14 | 57 | 2 | 20 | 46 | 22.78 | 47.61 |  |
| model_000_08 | -16.2 | 1.20E-12 | 10 | 17 | 35 | 5 | 39 | 59 | 22.64 | 47.58 |  |
| model_000_09 | -13.3 | 1.70E-10 | 21 | 13 | 53 | 3 | 12 | 45 | 22.56 | 47.72 |  |
| ICs (Inter-residue contacts) and NIS (Non-interacting surface) terms indicate the contribution of various types of residues to the overall binding affinity | | | | | | | | | | | |

^a^Data were calculated by using PRODIGY (PROtein binDIng enerGY prediction) server (https://rascar.science.uu.nl/prodigy/).

**Table S6. The interacting residues between ELF4 and TRIB3^a^**.

| Amino acid position of ELF4 | Amino acid position of TRIB3 |
| --- | --- |
| 10, 12 | 42, 149-151 |
| 19-54 | 62-75, 101-134 |
| 205-212, 257,271-277, 292 | 344-357 |
| 244-245, 261-263, 270, 280-286 | 174-177, 208-217 |
| 263, 270, 280, 282 | 234-240 |
| 563, 567 | 338-346 |
| 650-663 | 44-51, 219-232, 259-274 |

^a^The predictions were done by PRODIGYserver (https://rascar.science.uu.nl/prodigy/).

**Figures**


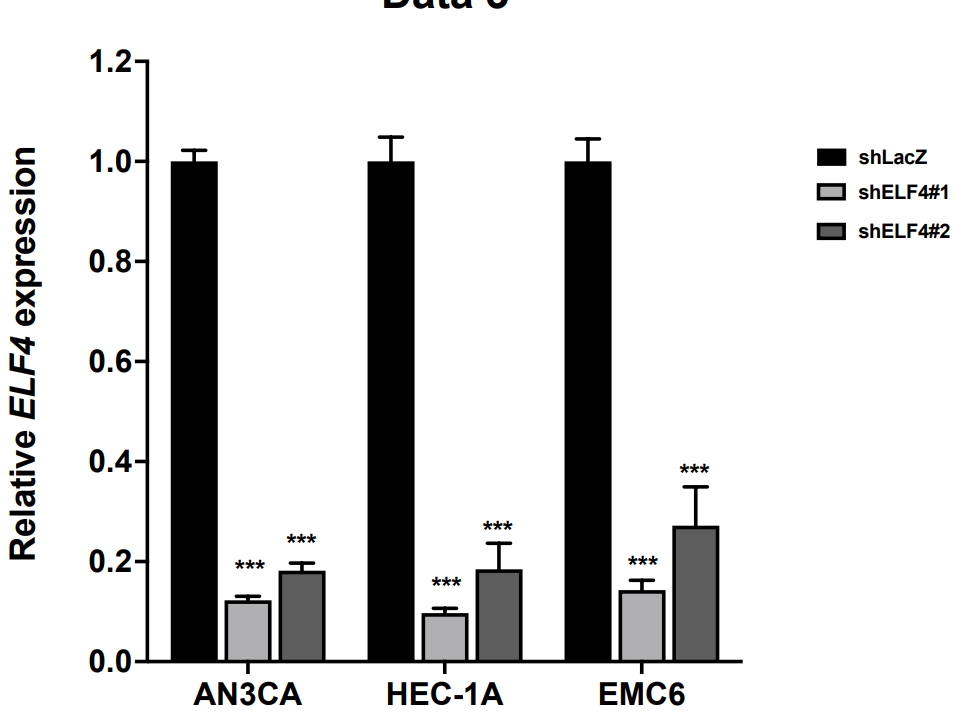


**Figure S1.** Knockdown efficiency of ELF4 shRNA in EC cells. AN3CA, HEC-1A and EMC6 cells were transduced with lentiviruses carrying shLacZ or ELF4-specific shRNAs and selected with 2 µg/ml puromycin for three days. Relative ELF4 mRNA levels were determined using SYBR Green-based qRT-PCR. Data are presented as fold changes (mean ± SD) relative to shLacZ from three individual experiments. **, p < 0.01, ***, p < 0.001 when compared to shLacZ.


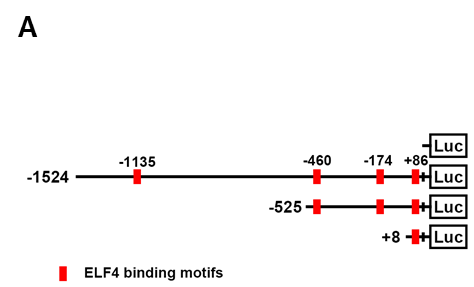


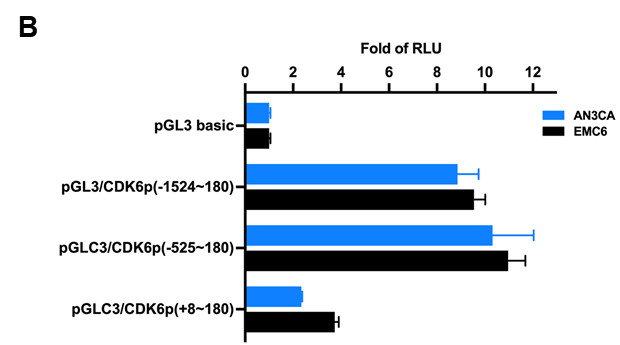


**Figure S2.** Human CDK6 promoter activity in AN3CA and EMC6 cells. (A) 5’ deletion constructs of the CDK6 promoter. The potential ELF4 regulatory regions were indicated as red bars. (B) AN3CA and EMC6 cells were transfected with pGL3-luciferase reporter plasmids containing CDK6 promoter constructs or pGL3-basic luciferase reporter for 48 h. Relative luciferase activity of pGL3-CDK6 constructs was normalized to pGL3-basic and expressed as fold changes (mean ± SD) relative to the pGL3-basic control from three independent experiments. Luciferase activities for AN3CA and EMC6 cells were shown in separate bars.

.


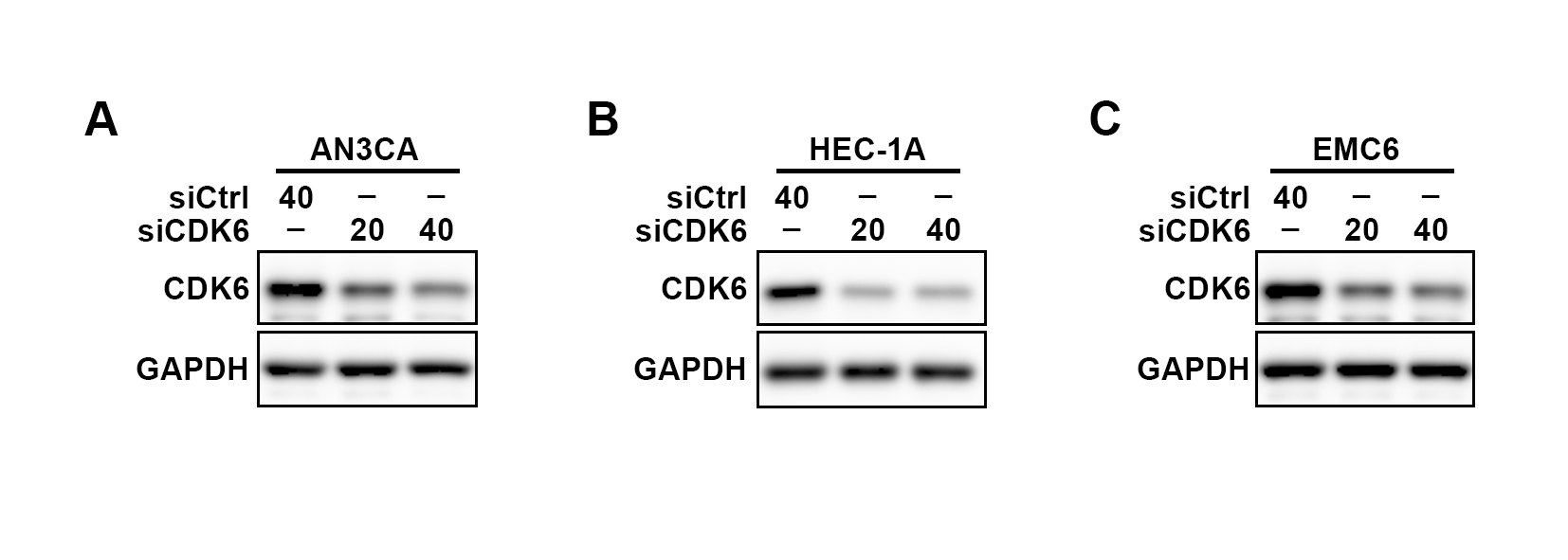


**Figure S3. The knockdown efficiency of CDK6 siRNA in EC cells.** AN3CA (A), HEC-1A (B) and EMC6 (C) cells were transfected with 40 nM of negative control siRNA (siCtrl), or 20 and 40 nM CDK6 siRNAs (siCDK6) for 24 hours using TransIT-X2 transfection reagent. The protein levels of CDK6 were determined by western blot analysis.


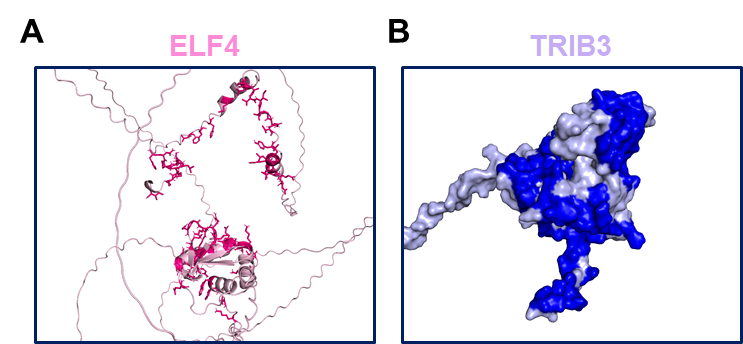


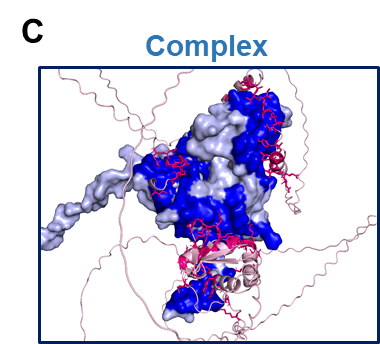


**Figure S4. Predicted interaction between ELF4 and TRIB3.** (A) and (B) depict the full-length protein structures of ELF4 and TRIB3, respectively, as predicted by AlphaFold. (C) illustrates a docking model of the predicted complex, with the TRIB3 interaction region highlighted in dark blue and the ELF4 interaction region in dark pink, against lighter shades for the full structures. Interaction regions were predicted based on high-confidence binding scores from AlphaFold.
