# Supplementary material for: ELF4/TRIB3/CDK6 Axis Promotes Cancer Stem Cell Activity in Endometrial Cancer
Source: J Cell Physiol. 2025 Nov 25;240(11):e70113. doi: 10.1002/jcp.70113 (PMC12645360; doi:10.1002/jcp.70113)
Supplement: Supplementary file 2 — IRB 114022. [file JCP-240-0-s003.pdf]

童綜合醫療社團法人童綜合醫院
Tungs' Taichung MetroHarbor Hospital

簡便行文表

☐極密件 ☐最速件
☐密件 ☑速件
☑普通件 ☐普通件

正　　本：急診醫學部陳俊佑科主任	發文單位：人體試驗委員會
副　　本：醫研部	聯 絡 人：楊月華
發文日期：2025年4月1日	分　　機：4635
附　　件：	

主旨：復台端申請之臨床研究計畫案審查結果，請 查照。

說明：

一、 台端申請之「E74 like ETS transcription factor 4對子宮內膜癌症惡化之分子機制探討」(IRB編號：114022)，經本院人體試驗委員會2025/04/01審核通過，符合研究倫理規範，至2026年3月31日止。

二、 請遵守本院規定：

1. 主持人應遵守隱私保護原則，妥善保管受試者資料。
2. 追蹤審查頻率為一年之研究案，計畫主持人於核准有效期前3個月(90天)內提出持續審查報告；追蹤審查頻率為半年之研究案，計畫主持人於核准有效期前6週 (45天)內提出持續審查報告；若未於期限內提出持續審查申請者，應立即停止試驗並申請結案。
3. 結案／撤案，請提出「結案審查」申請辦理「結案／撤案」
4. 試驗中止（終止）或變更時應檢送修正計畫向人委會提出申請，不能逕行變更計畫執行內容。
5. **IRB核准有效期間：2025/04/01~2026/03/31**。
6. **期中報告繳交期限：2026/03/31 以前**。
7. **結案報告繳交期限：2027/03/31 以前**。
8. 本會依衛生福利部規定不定期針對申請之研究計畫進行實地訪視，請計畫主持人於計畫開始執行時就應注意並隨時自我檢核。

三、 檢附「人體試驗委員會審查證明書」乙份及原計畫申請資料副本。

（副）主任委員：黃瑞斎

童綜合醫療社團法人童綜合醫院 人體試驗委員會 台中市梧棲區中棲路699號

童綜合醫療社團法人童綜合醫院

人體試驗委員會審查證明書

試驗名稱：E74 like ETS transcription factor 4 對子宮內膜癌症惡化之分子機制探討

本院主持人：急診醫學部陳俊佑科主任

共同主持人：中山醫學大學生物醫學科學系張文瑋教授

同意內容及版本：

1. 本院 IRB 編號：114022
2. 試驗計畫書編號：TTMHH-R1140032、版次：Version 2.0 / 2025-3-12
3. 受試者同意書版次：免除
4. 招募版次：無

定期繳交報告頻率：每年一次

IRB 核准有效期間：2025/04/01 至 2026/03/31 止

計畫預計執行期間：2025/04/01 至 2026/12/31 止

* 凡本院核准之臨床試驗／研究案，均應於有效日到期日前 3 個月至 6 週之前向本院人體試驗委員會提出申請延長本會核准期限，以取得本委員會同意以繼續執行試驗之證明。

* 逾期未通過持續審查申請者，應立即停止所有試驗活動及不可收納新受試者，直到通過持續審查取得核准函後始得繼續執行。

* 未依規定繳交報告或辦理計畫修正者，本會得中止該項臨床試驗，並拒絕受理該主持人往後之研究申請。

人體試驗委員會

（副）主任委員：黃瑞[illegible]

西元　2025　年　4　月　1　日
