# Supplementary figures and images for: ELF4/TRIB3/CDK6 Axis Promotes Cancer Stem Cell Activity in Endometrial Cancer
Source: J Cell Physiol. 2025 Nov 25;240(11):e70113. doi: 10.1002/jcp.70113 (PMC12645360; doi:10.1002/jcp.70113)

Figure 2

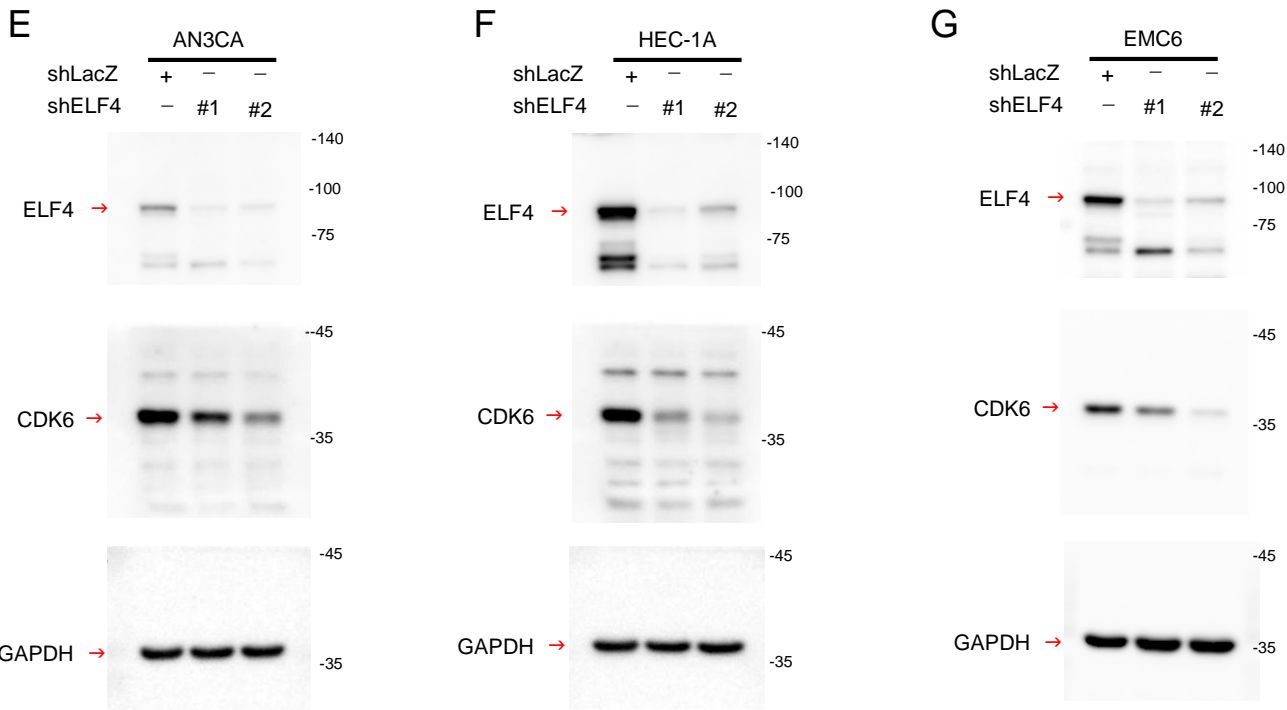

Figure 3

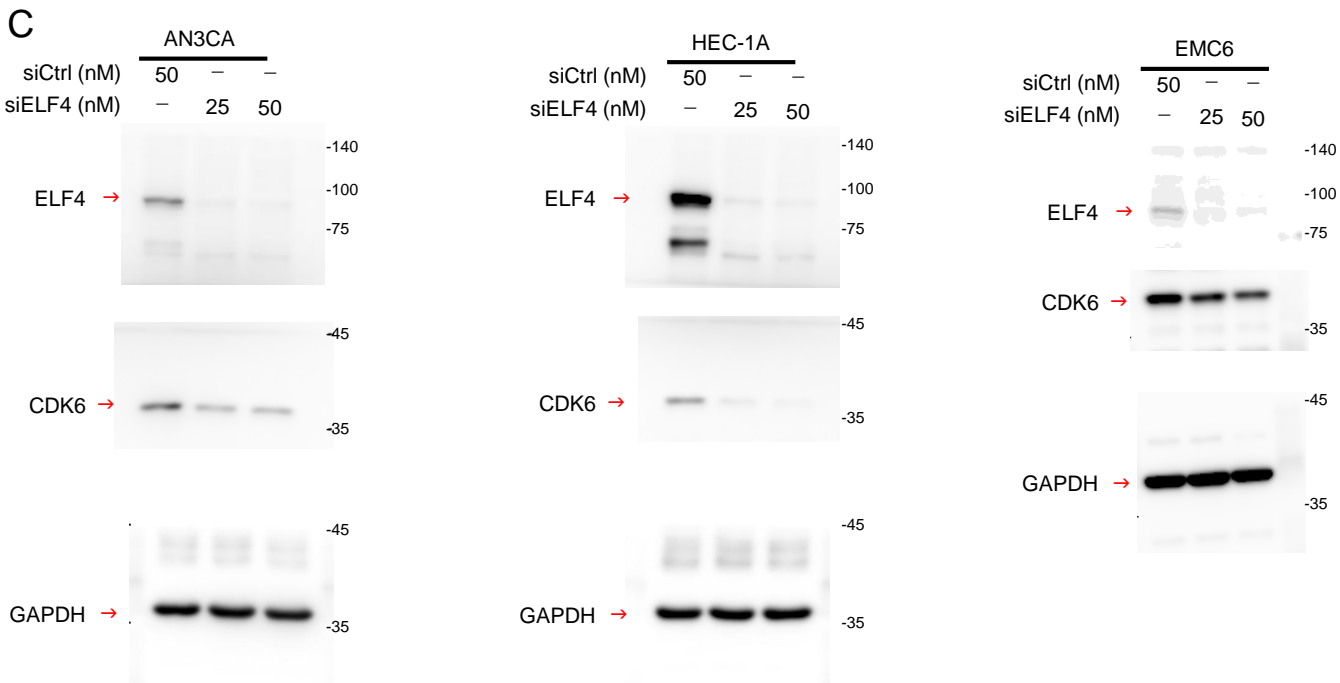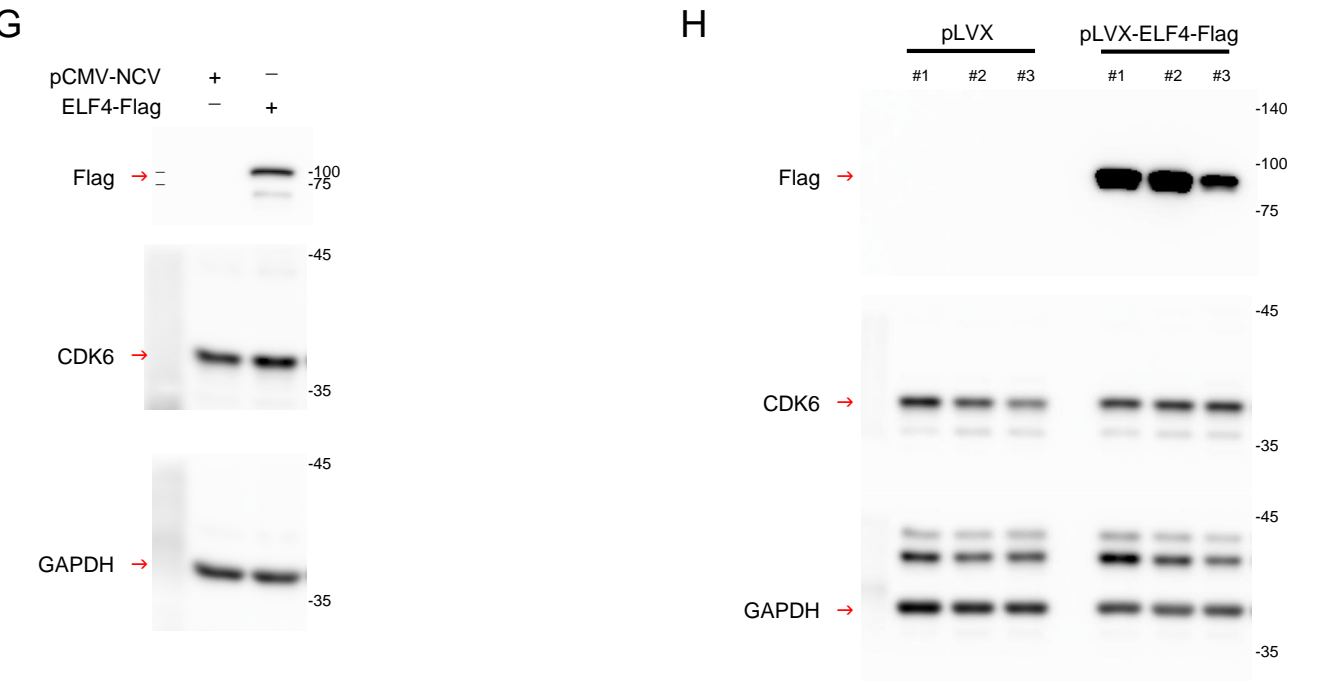

Figure 5

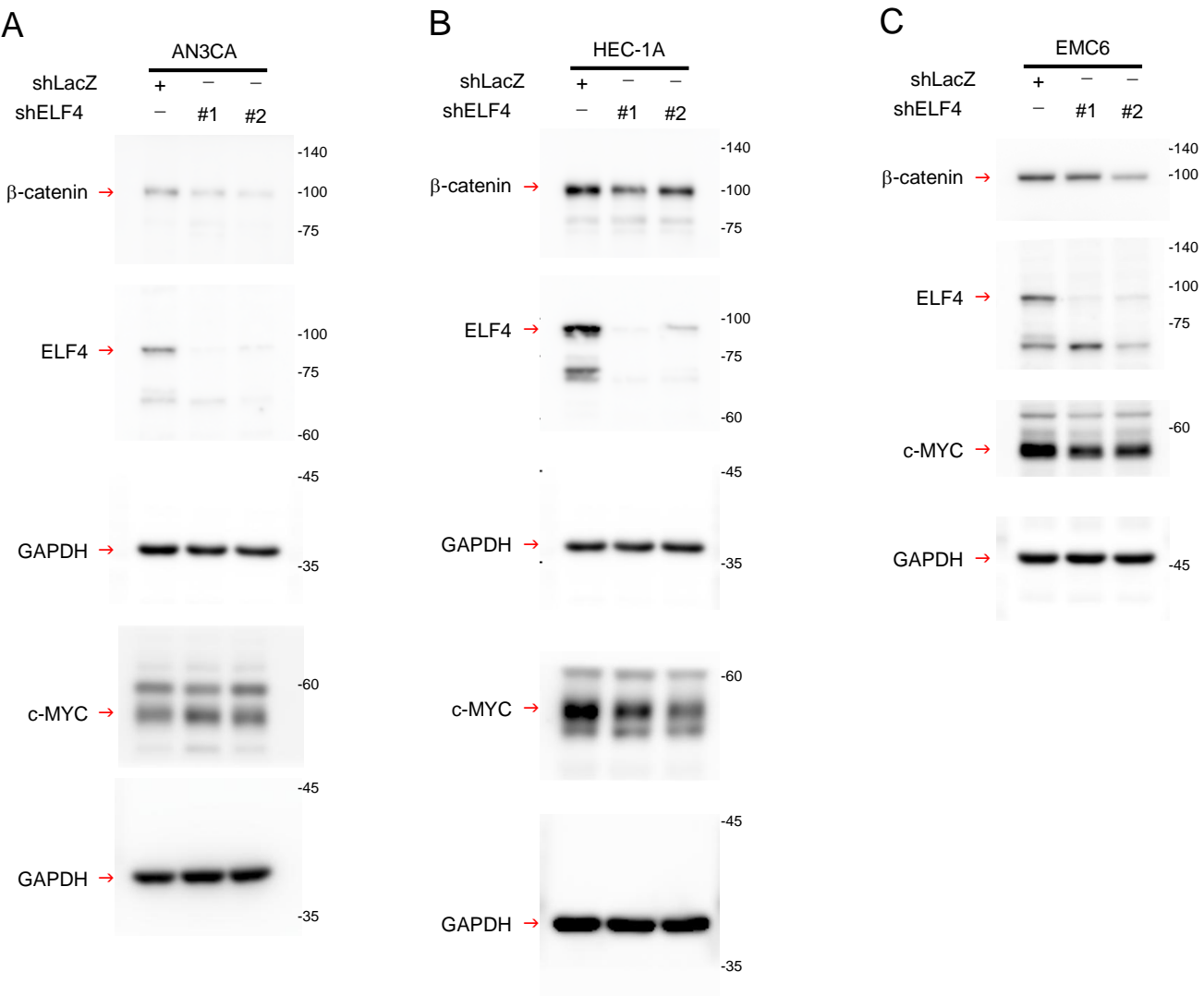

Figure 6D

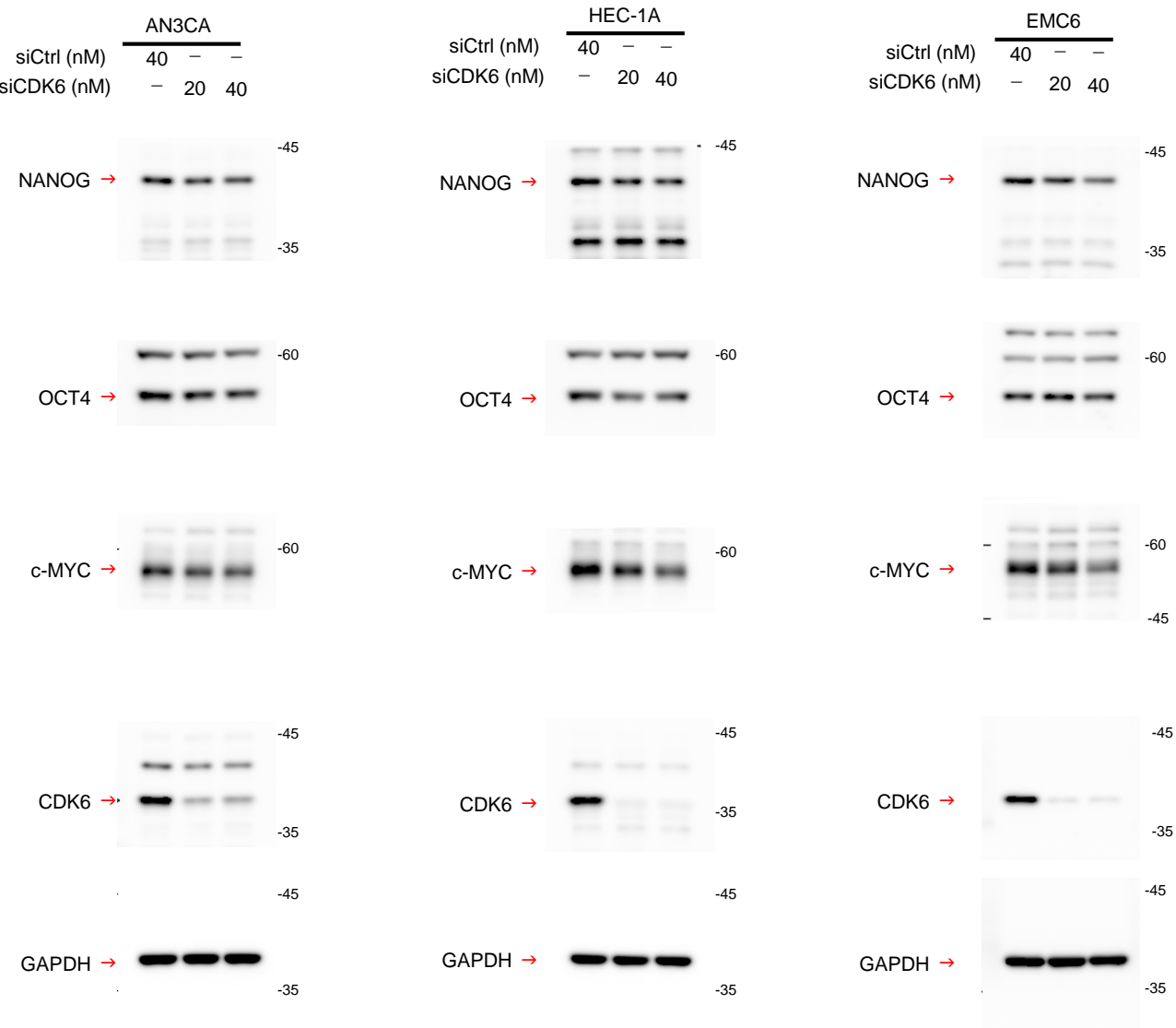

Figure 6H

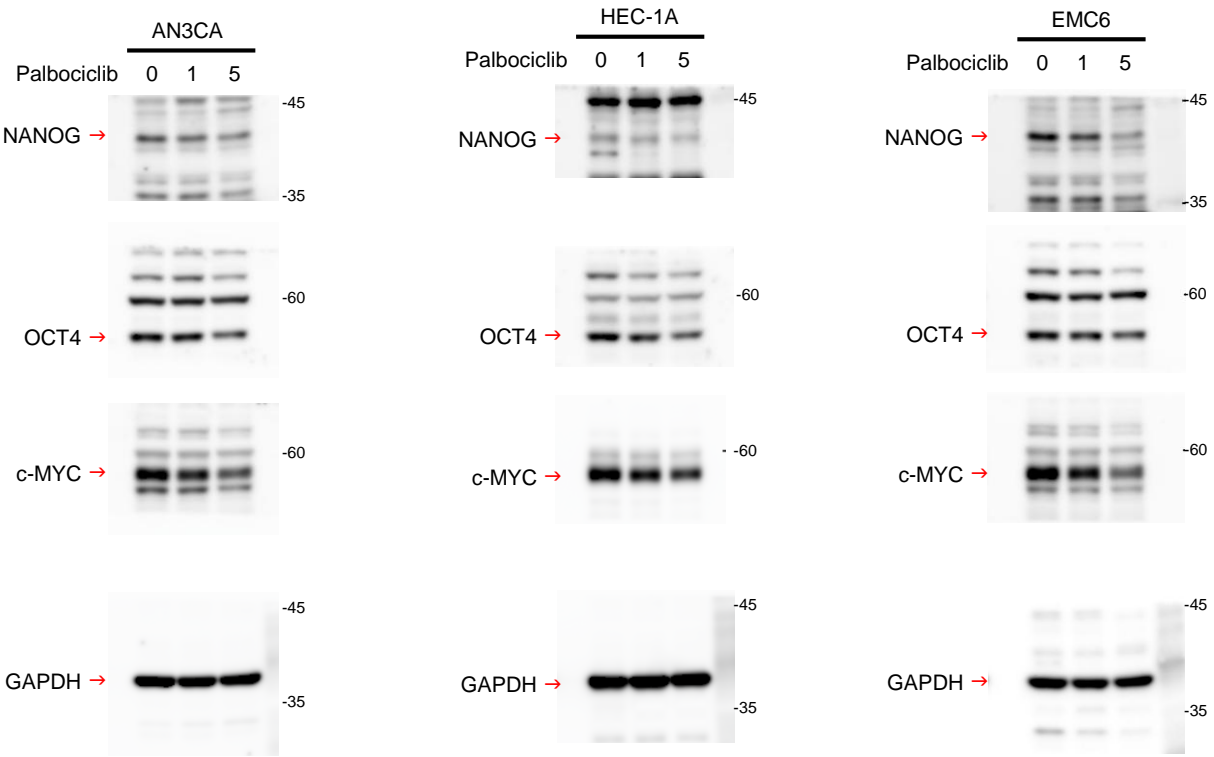

Figure 6J

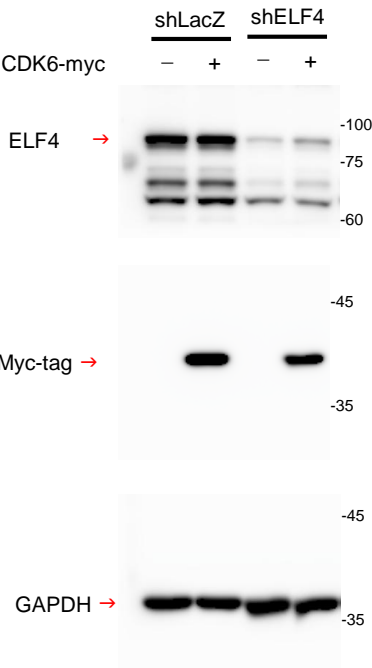

Figure 7D

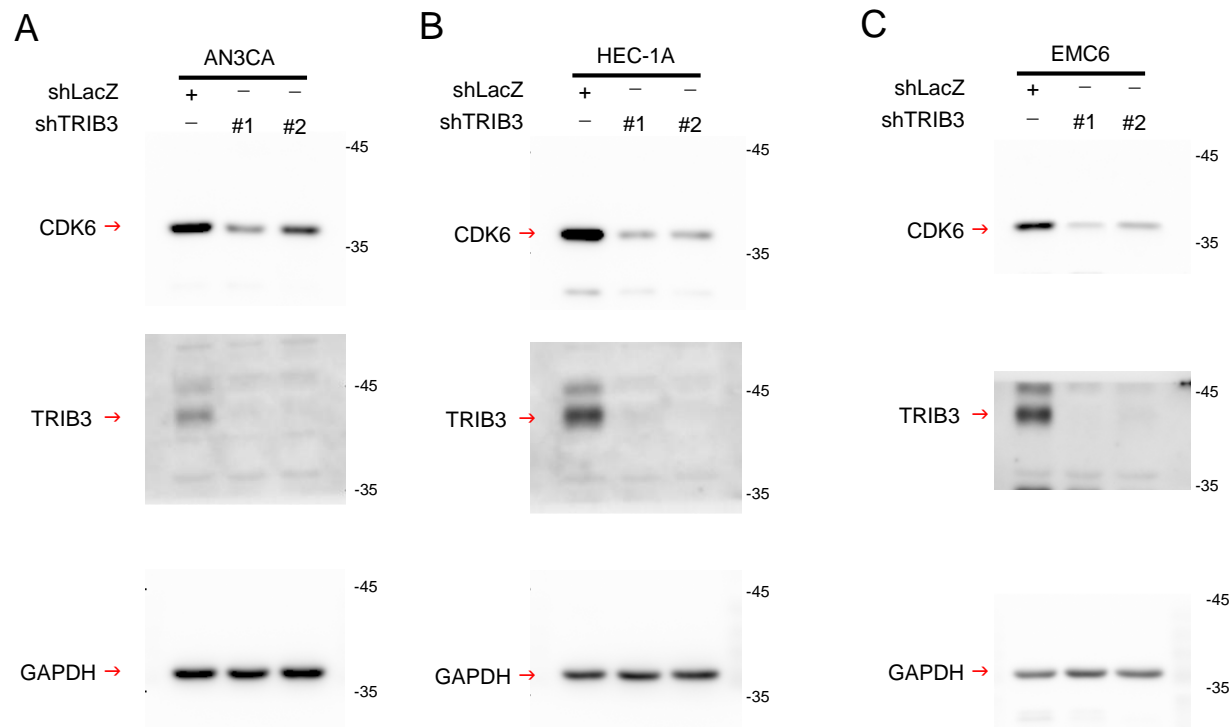

Figure 7G

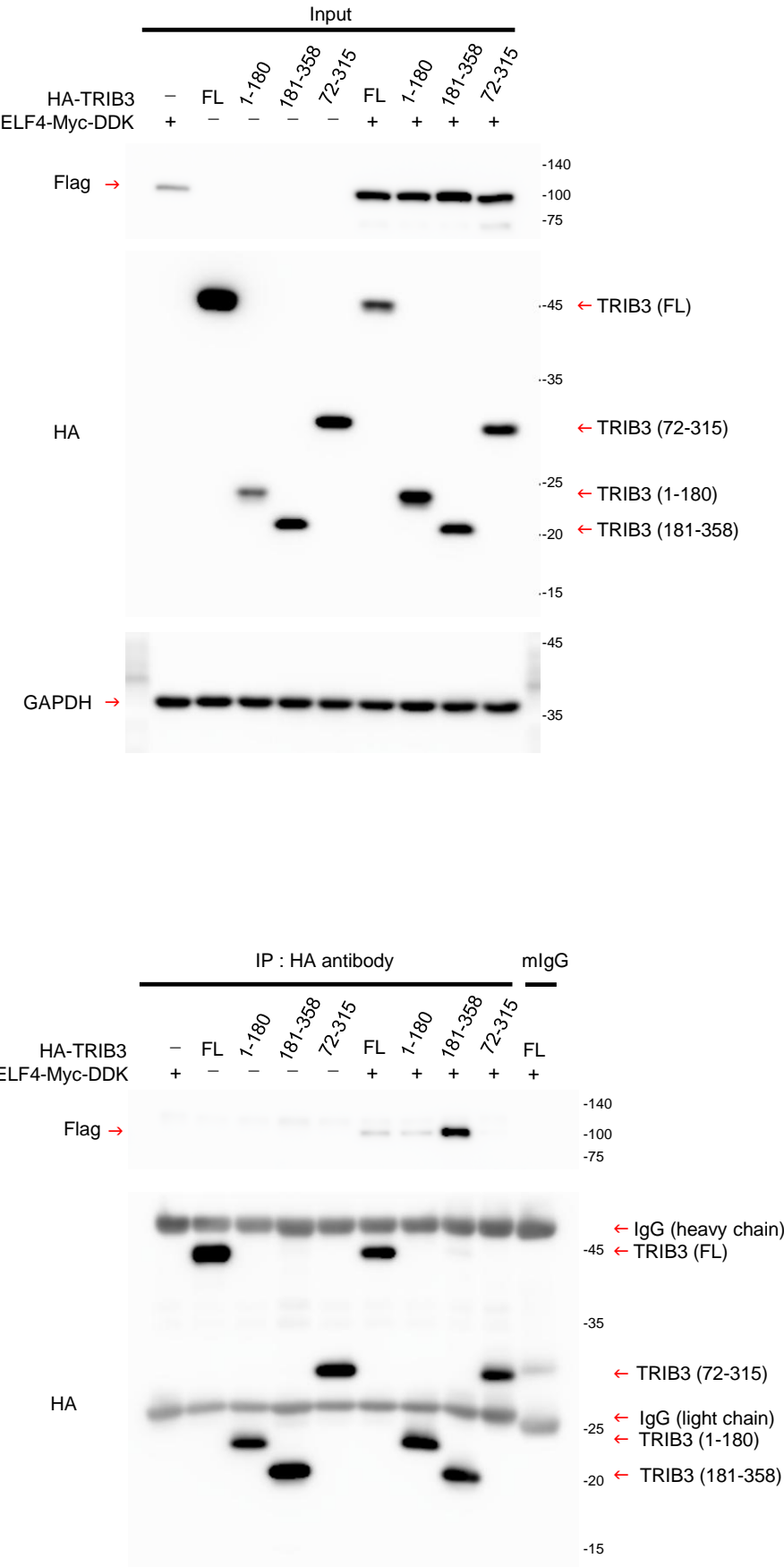

Figure 7H

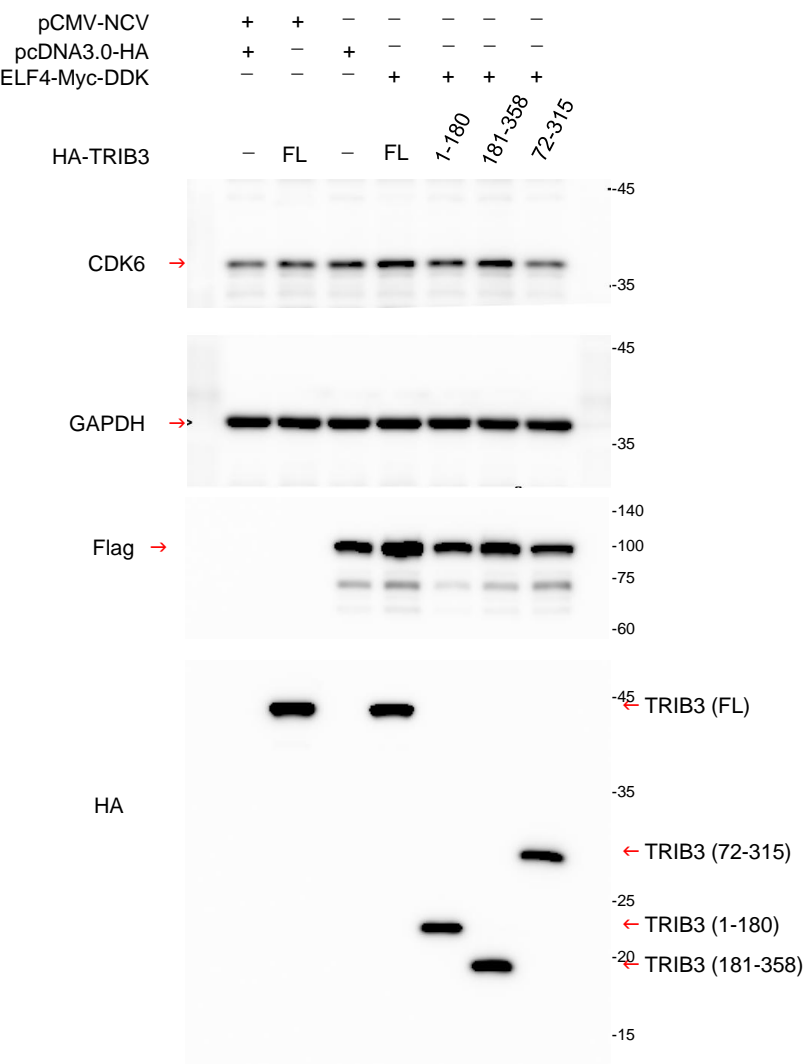

Figure 8D

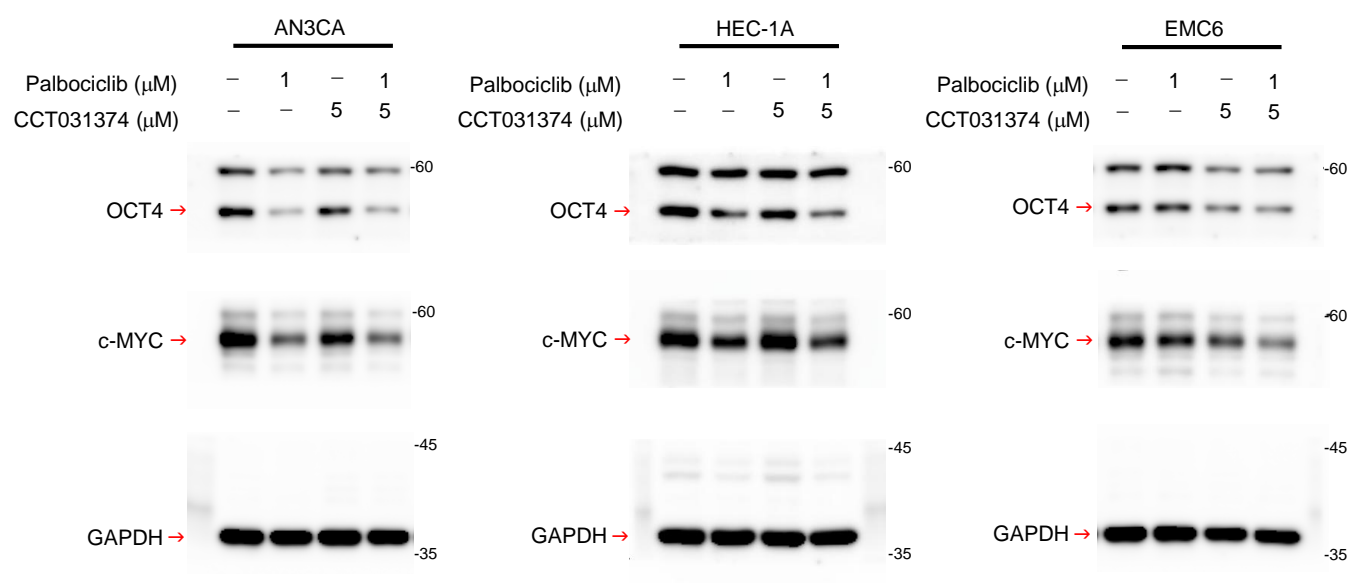

Figure 8F

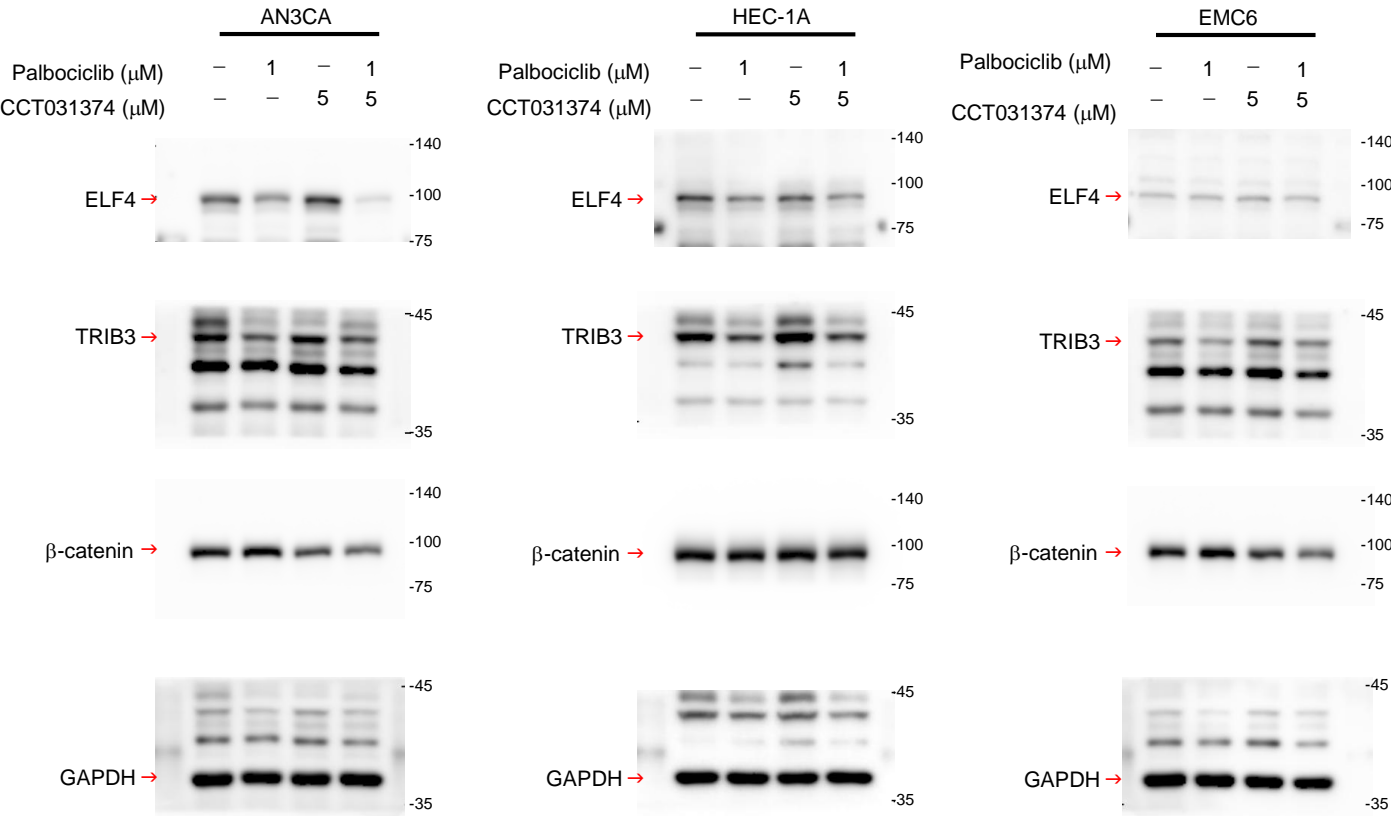

Supplement: Supplementary file 3 — western blot‐raw results_1031. [file JCP-240-0-s001.pdf]
